# Supramolecular fibrillation of peptide amphiphiles induces environmental responses in aqueous droplets

**DOI:** 10.1038/s41467-021-26681-2

**Published:** 2021-11-05

**Authors:** Richard Booth, Ignacio Insua, Sahnawaz Ahmed, Alicia Rioboo, Javier Montenegro

**Affiliations:** Centro Singular de Investigación en Química Biolóxica e Materiais Moleculares (CiQUS), Departamento de Química Orgánica, Universidade de Santiago de Compostela, 15705 Santiago de Compostela, Spain

**Keywords:** Self-assembly, Supramolecular polymers, Self-assembly

## Abstract

One-dimensional (1D) supramolecular polymers are commonly found in natural and synthetic systems to prompt functional responses that capitalise on hierarchical molecular ordering. Despite amphiphilic self-assembly being significantly studied in the context of aqueous encapsulation and autopoiesis, very little is currently known about the physico-chemical consequences and functional role of 1D supramolecular polymerisation confined in aqueous compartments. Here, we describe the different phenomena that resulted from the chemically triggered supramolecular fibrillation of synthetic peptide amphiphiles inside water microdroplets. The confined connection of suitable dormant precursors triggered a physically autocatalysed chemical reaction that resulted in functional environmental responses such as molecular uptake, fusion and chemical exchange. These results demonstrate the potential of minimalistic 1D supramolecular polymerisation to modulate the behaviour of individual aqueous entities with their environment and within communities.

## Abstract (web summary)

One-dimensional (1D) supramolecular polymers are commonly found in natural and synthetic systems but very little is currently known about the physico-chemical consequences and functional role of 1D supramolecular polymerisation confined in aqueous compartments. Here, the authors describe the different phenomena that resulted from the chemically triggered supramolecular fibrillation of synthetic peptide amphiphiles inside water microdroplets.

## Introduction

To understand and apply the different supramolecular designs found in biomolecular scaffolds, new strategies capitalise on synthetic surrogates that replicate their structure and function^1–5^. In particular, synthetic one-dimensional (1D) supramolecular biomaterials have shown great potential for their application in biomedicine and soft nanofabrication^6^. Some of these synthetic materials imitate the hierarchical self-assembly of biological building blocks across orders of magnitude in length, spanning from short nanotubes to microfibrillar networks^7,8^. This hierarchical assembly strongly amplifies the intramolecular and intermolecular non-covalent contacts along the resulting longitudinal assemblies^9–12^. Recent examples of such synthetic fibrillar biomaterials have demonstrated how these multivalent interactions can be used to transduce the hierarchical self-assembly of molecular entities into the macroscopic forces required to transform the size, shape and functional behaviour of soft matter^4,13,14^. In addition, new bottom–up strategies are being developed for the artificial reconstruction of different biomimetic tubular and fibrillar materials^14–16^. For example, the rheological properties of the cellular matrix have been recently mimicked, even with spatial resolution^17,18^, by hydrogelation of amphiphilic peptide nanotubes inside aqueous microdroplets^19,20^. 1D conductive supramolecular polymers, composed of artificial amino acids, can be prepared in situ in aqueous media by coupling a biocatalytic reaction with monomer nucleation and elongation processes^21^. Photo-responsive tubular assemblies, also made from synthetic amphiphiles in aqueous media, can encapsulate short nucleotides and control their duplex binding upon external light sources^22^. Exchange of chemical information^23^ between water droplets has been recently achieved using semipermeable membranes of amphiphilic triblock copolymers^24^.

In most of these synthetic assemblies, the hydrophobic effect constitutes a primary and ubiquitous driving force in aqueous media required to build ordered ensembles, such as micelles, bilayers, duplexes, coiled-coils, etc.^8,25,26^. However, beyond hydrophobicity, 1D anisotropic growth requires the additional contribution of hydrogen bonding and/or other directional non-covalent interactions that orient and stabilise the required supramolecular elongation in water^8,27^. Importantly, small differences in the number and/or position of these directional interactions are often sufficient to switch between different assemblies such as micelles, vesicles or fibres, with completely different supramolecular ordering and functional purposes^28,29^. Over the past years, the synthesis and assembly transitions of artificial amphiphiles have been carefully studied in the context of biomimetic compartmentalisation and autopoiesis^30–33^. Physical autocatalysis has been confirmed as a fundamental mechanism that amplifies monomer synthesis and self-assembly in many of these amphiphilic encapsulating systems^34,35^. However, very little is known about the potential functionality derived from the chemically triggered self-assembly of amphiphilic fibrillar networks inside aqueous compartments. In this context, we hypothesised that a synthetic peptide precursor, trapped inside water microdroplets, could readily assemble supramolecular fibres under confinement after its chemical connection to a hydrophobic tail of suitable length. Capitalising on the resulting hydrophobic enhancement, the new amphiphilic product could transition from a dispersed state to a fibrillar structure guided by directional H-bonding arrays that would be encoded in the dormant peptide precursor. This supramolecular transition and the subsequent bundling of the microfibrillar networks could lead to structural and functional biomimetic responses within this confined space and its surroundings.

Here we report the molecular and microscopic phenomena that resulted from the in situ generation of a peptide amphiphile via oxime condensation^36,37^ of two dormant fragments, which triggered a physically autocatalytic reaction that induced the assembly of a microfibrillar network. Under confinement, these two coupled processes resulted in a series of unpredicted responses by aqueous microdroplets with their environment. A spatial reorganisation of the fibrillar network was observed in confinement, which triggered the selective uptake of different exogenous chemicals inside the droplets. Intriguingly, it was also found that this fibre-mediated internalisation of chemicals modulates the coalescence and the exchange of cargoes between droplet populations. This cascade of chemical and physical events, emerging from a simple chemical reaction, suggests a potential role of minimalistic amphiphiles and their fibrillar assemblies in the functional behaviour of individual aqueous entities.

## Results

### Molecular design and fibrillation in solution

**Fig. 1:**
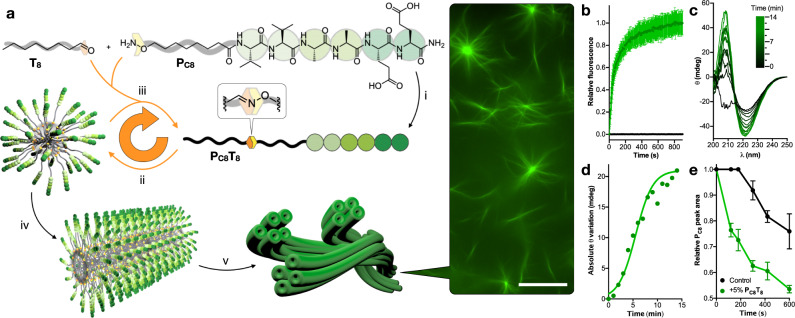
Autocatalytic formation of peptide-based microfibres. **a** Schematic showing: (i) precursor peptide **P**_**C8**_ reacting with octanal (**T**_**8**_) to form the peptide amphiphile product, **P**_**C8**_**T**_**8**_, though an oxime connection; (ii) micellar aggregation of the product **P**_**C8**_**T**_**8**_; (iii) co-assembly of **P**_**C8**_**T**_**8**_ with its precursors and the resulting physically autocatalytic behaviour (circular orange arrow); (iv, v) Hierarchical elongation into individual nanofibres and microfibrillar bundles, which can be seen by fluorescence microscopy stained with ThT. Scale bar = 20 µm. **b** Time-dependent ThT emission from an aqueous **P**_**C8**_ solution after addition of **T**_**8**_ (green) and without **T**_**8**_ (black); *n* = 3 (mean ± SD). **c** Time-dependent CD spectra of the reaction after addition of **T**_**8**_. **d** Change in CD signal (222 nm) during the reaction. **e** **P**_**C8**_ consumption in reaction with **T**_**8**_ (1 mM) either in the absence (control) or presence of pre-formed **P**_**C8**_**T**_**8**_ product (5% mol/mol) followed by HPLC (222 nm); *n* = 3 (mean ± SD).

We selected linear peptide amphiphiles as scaffolds for the assembly of fibrillar supramolecular architectures^36,38,39^. We employed a non-assembling peptide precursor, **P**_**C8**_, that could readily react with a hydrophobic tail, **T**_**8**_, to generate in situ the self-assembling peptide amphiphile, **P**_**C8**_**T**_**8**_ (Fig. 1a). This amphiphile is composed of two anionic glutamic acids, a short β-sheet-inducer sequence (two alanine and two valine residues) and an aliphatic chain at the *N*-terminus. In this case, the obvious synthetic disconnection between the pure peptide and aliphatic segments would require the use of a potential building block of tetradecanal, which would not be soluble in aqueous media. Instead, we designed the disconnection of **P**_**C8**_**T**_**8**_ into **P**_**C8**_ and **T**_**8**_, allowing the aqueous solubility and physiological compatibility of the two required molecular fragments^36^. Thus, the short non-fibrillating peptide precursor (**P**_**C8**_), bearing an alkoxyamine moiety, would react with octanal (**T**_**8**_) to yield the final oxime product (**P**_**C8**_**T**_**8**_) that would start the supramolecular polymerisation (Fig. 1a). The generation of this stable oxime connection will allow the functional interrogation of steady supramolecular **P**_**C8**_**T**_**8**_ fibres^38,40^.

The reaction between **P**_**C8**_ and **T**_**8**_ was first monitored in bulk water by fluorimetry using the probe Thioflavin T (ThT) to study reaction kinetics. The increase in ThT’s emission reports on the presence of peptide β-sheets^41^, as expected in the final assembled **P**_**C8**_**T**_**8**_ product. A rapid increase in ThT emission was observed upon addition of **T**_**8**_ to an aqueous solution of the peptide head (**P**_**C8**_), consistent with the formation of the corresponding supramolecular polymers (Fig. 1b). The transition from the latent peptide precursor to the final self-assembled product was also monitored by circular dichroism (CD), showing an exponential increase in the expected β-sheet-like signal as the reaction proceeds, which suggested a cooperative reaction-assembly mechanism (Fig. 1c, d). Indeed, the aqueous self-assembly of linear peptide amphiphiles, such as **P**_**C8**_**T**_**8**_, is driven by hydrophobic tail packing and β-sheet H-bonding between hydrophobic amino acids; Ala and Val in this case^38,40^. High-performance liquid chromatography (HPLC) analysis of the reaction kinetics showed a lag time preceding the formation of the final product (Fig. 1e and Supplementary Fig. 1). However, this delay was reduced by doping the reaction with the pre-formed product **P**_**C8**_**T**_**8**_ (5% mol ratio), which caused an approximate fourfold enhancement of the reaction rate (Fig. 1e). Epifluorescence microscopic imaging revealed the sudden emergence of long aster-like fibrillar networks, around 2 µm in diameter, 5 min after the addition of **T**_**8**_ to an aqueous sample containing the reactive peptide precursor **P**_**C8**_ (Fig. 1 and Movie 1). Electron microscopic analysis of **P**_**C8**_**T**_**8**_ samples showed the expected nanofibrillar morphology with progressive bundling into micron-sized 1D networks with increasing concentration, whereas no fibrillar structures were formed by the precursor **P**_**C8**_ (Supplementary Fig. 2). It should be noted that decreasing pH or increasing salt concentration of the aqueous medium will promote peptide fibrillation due to protonation or ionic shielding of the glutamic acid residues, respectively^36^. However, in this work, the external buffer conditions were not modified at any point to solely rely on the chemical trigger **T**_**8**_ to induce peptide fibrillation and potential functional responses.

The observed reaction-assembly profile (Fig. 1d, e) is consistent with the initial formation of micellar aggregates from the reaction product **P**_**C8**_**T**_**8**_, as evidenced by pyrene fluorescence titrations that showed a critical micellar concentration (CMC) of ca. 34 µM (Supplementary Fig. 3). From a supramolecular perspective, these micelles can establish a phase separation between an inner hydrophobic core and polar peptide shell, which drives the accumulation of precursor **P**_**C8**_ and **T**_**8**_ at either phase, thus increasing the reaction rate (Fig. 1a)^40,42^. Beyond the self-assembly of **P**_**C8**_**T**_**8**_ at its CMC, the reaction product displayed a second supramolecular transition at ca. 200 µM, which would correspond to the elongation of micelles into fibres (Supplementary Fig. 3). The precursor **P**_**C8**_ did not show any evidence of micelle formation with this same pyrene assay, even at concentrations as high as 1 mM. However, a 12-fold ThT emission increase was observed in **P**_**C8**_ samples (1 mM) doped with sub-fibrillar concentrations of **P**_**C8**_**T**_**8**_ (20–150 µM), demonstrating the co-assembly of the precursor and product (Supplementary Fig. 4)^40,42^. Taken together, these experimental observations were consistent with a physical autocatalytic reaction mechanism, which is triggered by the phase separation at micellar and fibrillar interfaces and the co-assembly of precursors and products (Fig. 1a)

### Confined fibrillation within aqueous droplets

**Fig. 2:**
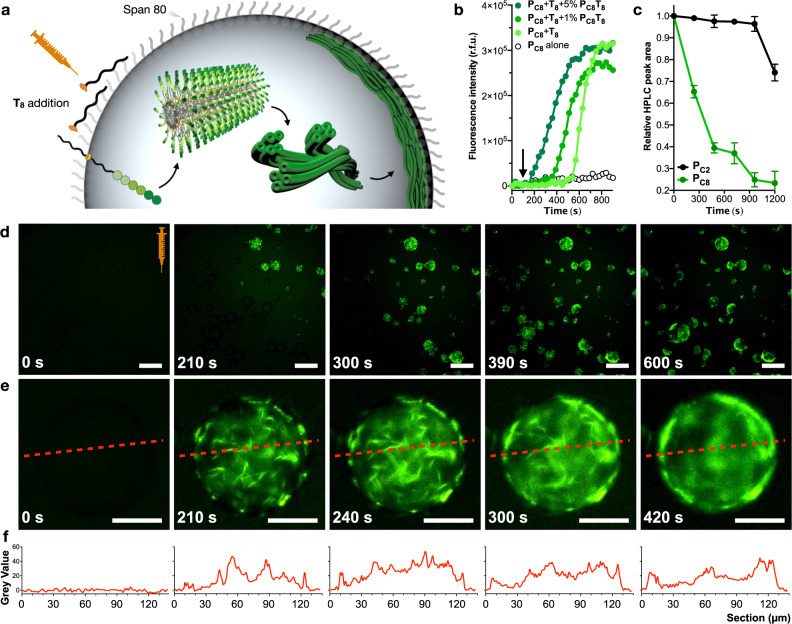
Physical autocatalysis in water-in-oil emulsion. **a** Schematic showing the autocatalytic fibre-forming reaction in aqueous droplets. Dormant **P**_**C8**_ precursor loaded into the microdroplets reacts with **T**_**8**_ added to the external oil phase, thus triggering **P**_**C8**_**T**_**8**_ production and fibrillation, ultimately generating cortical microfibrillar bundles at droplet interfaces. Note all droplets in this manuscript contain the surfactant Span 80. **b** Time-dependent ThT emission in the presence of the confined reaction between **P**_**C8**_ and **T**_**8**_ doped with their product **P**_**C8**_**T**_**8**_ (1–5% mol, dark greens), no doping (light green) and negative reaction control without **T**_**8**_ (white). Octanal (**T**_**8**_) is added to the samples after 100 s (arrow). **c** Plots showing the competition between **P**_**C8**_ (green) and **P**_**C2**_ (black) in droplets; data collected by HPLC (222 nm); *n* = 3 (mean ± SD). **d**, **e** Fluorescence micrographs showing diffusion-dependent reaction after addition of **T**_**8**_. The syringe indicates the point of addition of **T**_**8**_. Scale bars = 200 µm (**d**) and 50 µm (**e**). **f** Intensity profiles of droplets shown in **e**.

Having established that the reaction proceeds by physical autocatalysis across the interface of amphiphilic assemblies, the **P**_**C8**_ peptide was confined inside aqueous droplets of a water-in-oil (w/o) emulsion to test whether the reaction and subsequent self-assembly would tolerate an additional interfacial boundary (Fig. 2a). Reaction kinetics were first monitored by fluorescence spectroscopy in the presence of ThT, showing a lag time followed by an abrupt increase of the probe’s emission (Fig. 2b). This emission profile implies the successful reaction between **P**_**C8**_ and **T**_**8**_ followed by the self-assembly process. As expected, the lag phase observed was gradually shortened when doping the droplets with increasing concentrations of the final product **P**_**C8**_**T**_**8**_ (Fig. 2b)^43^. Competition experiments were carried out in the presence of a second reactive peptide bearing a shorter two carbon tail (**P**_**C2**_). Thus, **P**_**C8**_ and **P**_**C2**_ were mixed in equal amounts inside the water droplets and reacted against each other by addition of **T**_**8**_. HPLC analysis confirmed the faster reaction of the peptide precursor **P**_**C8**_ compared to its shorter analogue **P**_**C2**_, as it could be expected from the less amphiphilic structure of **P**_**C2**_ and its product, **P**_**C2**_**T**_**8**_, which is unable to self-assemble into fibres^36^ (Fig. 2c).

To image the confined fibrillation process, water droplets containing the peptide precursor **P**_**C8**_ and ThT were placed on a microscopy slide and octanal (**T**_**8**_) was added at the top-right end of the emulsion for it to diffuse and react across the slide (Fig. 2d–f and Movie 2). After approximately 3 min, microfibrillar structures emerged from the lumen of the droplets and distributed homogeneously across the whole aqueous compartment (Fig. 2d). Intriguingly, as the fibrillar networks grew longer and bundled together, fibres reorganised spatially from the core to the cortex of the water droplets (Fig. 2e, f and Movie 3). Confocal fluorescence microscopy confirmed the accumulation of fibres at the cortex of the droplets by the end of the fibrillation–migration process, as found in *z*-stacked slices of droplets (Movie 4). As shown by pixel quantified time-lapse analysis, **P**_**C8**_**T**_**8**_ fibrillation originates at both the interior and border of droplets at a very similar pace, but after approximately 1 min, ThT emission in the edge starts increasing at the expense of that in the centre (Supplementary Fig. 5, *t* ~ 240 s), indicative of the outward movement of the fibres towards the border of the droplets. It is expected that free-floating **P**_**C8**_**T**_**8**_ fibres, in random motion due to molecular thrust during self-assembly^44,45^, tend to accumulate at droplet boundaries with high local concentration of **P**_**C8**_**T**_**8**_ monomers (vide infra surface tension assay), which should drive their elongation and bundling into larger assemblies at droplet edges.

**Fig. 3:**
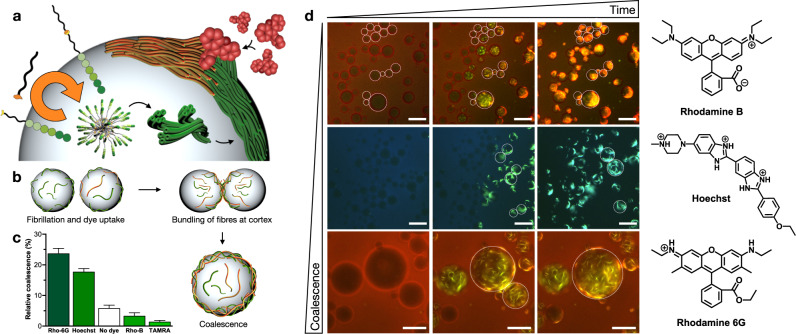
Fibre-mediated uptake of external molecules and droplet coalescence. **a** Schematic showing fibre-forming autocatalytic reaction in a w/o emulsion triggering the uptake of external dyes. Note that Span 80 has not been drawn for clarity. **b** Schematic showing dye-mediated coalescence of adjacent droplets induced by confined fibrillation. **c** Relative coalescence of droplets undergoing fusion in the presence of external dyes (*n* ≥ 65, see Supplementary Fig. 10). These values are calculated as the relative increase (%) in average droplet diameter after the addition of **T**_**8**_ (see Supporting Information). **d** Fluorescence micrographs showing coalescence of adjacent droplets in the presence of different external dyes (see structures on the right). Contoured droplets are representative positive/negative coalescence examples. Scale bars = 80 µm. All experiments contain ThT at 5 µM.

Once found that confinement allowed the physically autocatalytic process and triggered a spatial reorganisation of the microfibres within the water microdroplets, we focussed our attention on the potential consequences that these coupled chemical and physical processes could have on the droplets. It was observed that, after fibrillation, droplets in proximity tended to maximise their physical contact with other droplets and showed stochastic coalescence events that mixed the internal content of the fused droplets (Fig. 3b and Movie 5). This coalescence, triggered by the formation of **P**_**C8**_**T**_**8**_ and its supramolecular polymerisation, can be assigned to the bundling of fibrillar networks at the cortex of adjacent droplets, resulting in the mixing and eventual fusion of the aqueous compartments (Movies 2 and 5). Dynamic surface tension^46^ measurements showed that both the peptide precursor and the final amphiphile decreased the surface tension of the water phase (Supplementary Fig. 6), an effect that should stabilise the emulsion droplets^47,48^, thus ruling out variations in surface tension as the driving force for droplet coalescence. It should be noted that the surfactant Span 80 was employed in all water droplet experiments to stabilise the emulsions (Fig. 2a). To investigate any potential interaction between this surfactant and the fibrillar assemblies, the β-sheet signal of **P**_**C8**_**T**_**8**_ fibres was monitored by CD with increasing concentrations of Span 80, which showed nearly identical profiles for all cases (Supplementary Fig. 7). In addition, no morphological differences could be noticed in the bundling fibres within droplets or in the bulk buffer (Figs. 1a and 2e), further suggesting no significant interference of the non-ionic surfactant Span 80 in the growth and bundling of the supramolecular fibres (Fig. 2a).

### Fibrillation-induced molecular uptake and coalescence

To further explore the interaction of droplets with their environment, the fibrillation process was studied in the presence of exogenous fluorescent probes possessing different physico-chemical properties (Fig. 3). Fibrillation experiments were initially performed in the presence of cationic Rhodamine 6G, which was quickly taken up inside droplets by the anionic fibrillar network (Fig. 3d). Surprisingly, in addition to the uptake of this dye, an increase in the rate of droplet coalescence was observed compared to samples in the absence of any dye (Supplementary Fig. 8 and Movies 6a, b). This intriguing behaviour can be explained by the neutralisation of anionic **P**_**C8**_**T**_**8**_ fibres by this cationic dye, which shields charge repulsions between fibrillar networks and thus promotes the bundling of neighbouring fibres and droplet fusion (Fig. 3b). Indeed, fibrillation experiments in the presence of the zwitterionic Rhodamine B dye showed efficient uptake of this dye but very limited droplet coalescence, reinforcing the role of the exogenous cargo to modulate fibre neutralisation and droplet coalescence (Fig. 3d, Movies 7a, b and Supplementary Fig. 8). An alternative cationic dye, Hoechst, which binds to the minor groove of double-stranded DNA, confirmed the enhancement in droplet coalescence when the supramolecular fibres capture an oppositely charged molecule (Fig. 3d, Supplementary Fig. 8 and Movie 8). Finally, the anionic dye TAMRA was explored as negative coalescence control of the proposed charge neutralisation principle. As expected, this anionic dye showed a slight reduction in the coalescence degree of fibrillating droplets below the number of original events observed in the absence of any external dye (Fig. 3c, Supplementary Fig. 8 and Movie 9). Pixel intensity analysis allowed the estimation of dye uptake, revealing higher uptake values for more cationic dyes: Hoechst ~ Rhodamine 6G > Rhodamine B > TAMRA (56% ~ 52% > 18% > 5% uptake; Supplementary Fig. 9). The extent of dye uptake follows the same trend as found for dye-mediated droplet coalescence (Fig. 3c and Supplementary Fig. 10), in which cationic dyes (i.e. Rhodamine 6G and Hoechst) induce fusion into larger droplet populations after anionic fibrillation, supporting the electrostatic foundation of fibrillar functionality.

### Enzymatic cascade across droplet populations

**Fig. 4:**
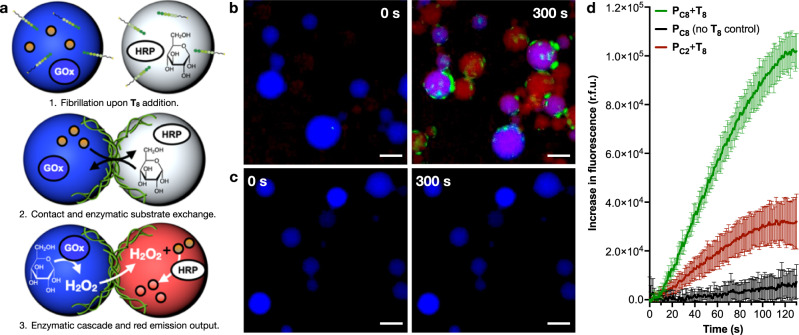
Microfibre-mediated exchange of chemical contents between droplets to trigger a biocatalytic pathway. **a** Scheme illustrating the steps involved in content exchange between droplets and resulting enzymatic reactions. The non-emissive Amplex Red precursor (orange dots) is converted into red emissive resorufin (red dots). Note Span 80 has not been drawn for clarity. **b**, **c** Fluorescence micrographs before (0 s) and 300 s after the addition of octanal (**T**_**8**_). Experiments carried out in the presence (**b**) and absence (**c**) of the aldehyde precursor **T**_**8**_. Scale bars = 70 µm. All experiments contain ThT at 5 µM. **d** Bulk red emission of emulsions containing both droplet populations, either in the presence (red) or absence (black) of **T**_**8**_, corresponding to a fibrillating and non-fibrillating system, respectively. The red profile shows a control sample with both droplet populations loaded with **P**_**C2**_, whose product **P**_**C2**_**T**_**8**_ is unable to self-assemble into fibres^36^; *n* = 3 (mean ± SD).

Being able to trigger molecular uptake by confined supramolecular fibrillation, the possibility of inducing contact-based exchange of cargoes between droplet populations was envisioned^24,49^. ThT emission profiles along droplet contacts revealed the preferential accumulation of fibrillar bundles at the interface between contacting droplets (Supplementary Fig. 11). Such fibre focalisation results from a partial inter-droplet bundling of fibres, which would generate tight contacts between neighbouring droplets to maintain their association and promote content exchange (Fig. 4a). To test this hypothesis, a two-step enzymatic cascade was incorporated into droplets, making substrate and product exchange between droplet populations necessary to generate a fluorogenic reporter at the end of the biocatalytic pathway (Fig. 4a). Therefore, catalysts and substrates of the enzymatic cascade between glucose oxidase (GOx) and horseradish peroxidase (HRP) were orthogonally encapsulated into two different populations of droplets^50^. In this coupled pathway, HRP uses H_2_O_2_, which is produced by GOx from glucose, to oxidise Amplex Red into fluorescent resorufin (red). DyLight 405 (blue) was attached to GOx to differentiate and visualise this droplet population. The two populations were mixed and **T**_**8**_ was added to the emulsion to initiate the fibrillation reaction while monitoring by fluorescence microscopy (Fig. 4b and Movie 10a). The appearance of red fluorescence in the HRP droplets confirmed the completion of the biocatalytic pathway and hence the exchange of cargoes between the two droplet populations. HRP droplets (colourless) in proximity to GOx droplets (blue) showed a fast increase in red fluorescence (Fig. 4b). The red emission found in blue GOx droplets confirmed the two-way exchange of substrates (i.e. glucose and Amplex Red) and products (i.e. H_2_O_2_ and resorufin) between populations, while macromolecular cargoes like blue-tagged GOx remained unexchanged (Fig. 4b and Movies 10a, b).

Red resorufin emission was observed at short reaction times before microfibrillation due to the assembly of short peptide 1D nanofibres, which could not be resolved by epifluorescence microscopy. The formation of these shorter 1D nanofibres was confirmed to occur right after **T**_**8**_ addition and to progressively bundle into larger microfibres with increasing reaction times (Supplementary Fig. 12). Individual droplet analysis demonstrated that populations in direct contact via cortical fibre clusters generate faster and more intense red emission than distant droplets (Movie 10b and Supplementary Fig. 12c). Control experiments in the absence of the fibrillation trigger **T**_**8**_ did not show any red-emitting droplets, demonstrating the role of the peptide fibres as transport and communication promoters between droplets (Fig. 4c and Movie 11). It must be noted that emulsions are dynamic systems that can inherently exchange material across their discontinuous phase, a phenomenon that is promoted in the presence of amphiphilic molecules^51,52^. However, a control experiment with a non-fibrillating yet amphiphilic analogue, **P**_**C2**_**T**_**8**_^36^, showed a threefold lower red emission than **P**_**C8**_**T**_**8**_, supporting the role of supramolecular fibres in enhancing the exchange of chemicals between droplet communities (Fig. 4d). Combining all experimental evidence, we propose a dual synergistic role of **P**_**C8**_**T**_**8**_ in promoting droplet communication: (i) fibrillar bundles across droplet interfaces establish tight contacts that might generate local points of phase continuity, allowing the direct exchange of aqueous content (Supplementary Fig. 11); (ii) the amphiphilic nature of **P**_**C8**_**T**_**8**_ can increase chemical transport and droplet cross-talk^51,52^, which would also be reinforced by the fibre-mediated clustering of droplets.

## Discussion

The application of synthetic supramolecular polymers is gaining momentum to mimic, understand and adjust the complex functional behaviour of biomolecular assemblies^13,53–58^. In addition to the design of new functional synthetic systems, the artificial reconstitution of confined tubular networks can trigger the transformation of soft materials, as exemplified by shape deformations^59^, budding fissions^60,61^ and stabilisation against coalescence^62^, among others. The objective of this work was to study the unexplored potential functional behaviour of water droplets, which could be triggered by the chemical reaction of confined synthetic precursors that self-assemble into supramolecular 1D networks. To this end, we employed a suitable synthetic disconnection of a linear peptide amphiphile to allow the aqueous compatibility of its hydrophobic and directional hydrogen bonding blocks. The aqueous oxime condensation of these precursors rapidly generates in situ a self-assembling amphiphile that drives the accumulation of the corresponding hydrophobic and polar precursors by co-assembly, thus self-catalysing the formation of assembling product and its supramolecular propagation^63,64^. The system could be incorporated inside aqueous droplets, allowing the study of these fibrillar networks under confinement, which could trigger a series of coupled environmental responses. We show that the continuous growth and bundling of supramolecular fibres and their confined spatial rearrangement can trigger the uptake of exogenous substances by the droplets, which in turn can control the morphological transformations, the coalescence and the exchange of chemicals in droplet populations. The series of coupled chemical and physical events reported here, which emerge from a single reaction, suggests that chemically triggered 1D self-assembly can play a critical role in the functional properties of individual aqueous entities.

Elongated structures and fibrillar networks are commonly identified in cells and in the semiliquid protoplasm^65^. Intriguing discoveries on primitive fibrillar networks have confirmed the presence of different early supramolecular biopolymers that were involved in functional processes such as the emergence of basic phagocytic behaviour and perhaps also in symbiotic engulfment^66^. However, experimental confirmation of such functional responses from simple synthetic supramolecular precursors has remained elusive. The experiments reported here constitute a first proof of principle that 1D hierarchical self-assembly can indeed trigger coordinated complex functional behaviour between individual aqueous entities (i.e. physical autocatalysis, self-assembly, uptake, coalescence and exchange of materials) without the need of a complex molecular machinery. It also transpires from these results that minimalistic 1D supramolecular polymerisation plays a role in the functional mechanisms that individual aqueous entities may have used to interact with their environment and within communities. Overall, these results will contribute to the bottom–up design and basic understanding of more complex 1D supramolecular biomimetic assemblies and the future development of new 1D functional materials with life-like properties and stimuli-responsive behaviour.

## Methods

### Peptide head (P_C8_ and P_C2_) synthesis

Peptide heads were synthesised by standard Fmoc solid-phase peptide synthesis from Rink amide resin. Coupling steps employed Fmoc-protected amino acid (3 equiv.), HBTU (2.8 equiv.) and DIEA (4 equiv.) in dimethylformamide (DMF) for 30 min. Fmoc removal steps employed 20% v/v piperidine in DMF for 10 min. After the final Fmoc deprotection, 8-[Boc](aminooxy)]octanoic acid or (Boc-aminooxy)acetic acid (2 equiv., see ESI for synthetic protocol and characterisation) was coupled to the *N*-term in the presence of HBTU (1.8 equiv.) and DIEA (3 equiv.) for 1 h to obtain **P**_**C8**_ or **P**_**C2**_, respectively. The resin was then cleaved with TFA:DCM:TIS:H_2_O (90:5:2.5:2.5%v/v) for 2 h and the afforded solution precipitated into chilled diethyl ether. The pellet obtained was purified by preparative HPLC (C18) using a 0:100–50:50 gradient of CH_3_CN:H_2_O + TFA (0.1%v/v) and the pure fractions concentrated and freeze-dried. Peptide heads were thus obtained as white powders: **P**_**C8**_ = 29.4 mg (38%) and **P**_**C2**_ = 21.1 mg (31%).

### Peptide amphiphile (P_C8_T_8_ and P_C2_T_8_) synthesis

Peptide amphiphiles were synthesised by addition of octanal (**T**_**8**_, 1.1 equiv.) to either peptide head (**P**_**C8**_ or **P**_**C2**_, 1 equiv.) in dimethyl sulfoxide (5 mg mL^−1^ of peptide head) and the reaction was shaken at 60 °C until completion by HPLC–mass spectrometry. The solution was precipitated three times in cold diethyl ether, dissolved in water and lyophilised to obtain the corresponding white powders: **P**_**C8**_**T**_**8**_ = 5.5 mg (96%) and **P**_**C2**_**T**_**8**_ = 5.1 mg (91%).

### Synthesis and self-assembly of P_C8_T_8_ fibres in bulk water

Stock aqueous solutions of **P**_**C8**_ (40 μL, 10 mM), MES buffer pH 6.0 (40 μL, 500 mM) and ThT (40 μL, 100 mM) were mixed with 280 μL of Milli-Q water and octanal (**T**_**8**_) was added (5 μL, 405 mM in dodecane). Reaction and self-assembly were followed by HPLC (see ESI 2.6.), fluorescence spectroscopy (ThT: ex = 450 nm; em = 482 nm), epifluorescence microscopy (ThT: ex = 480/30 nm, em = 535/45 nm) and CD. Reaction volumes could be scaled up to 1 mL. Seeded (auto-catalysis) experiments contain the pre-formed product (mol%) before **T**_**8**_ addition.

### Confined synthesis and self-assembly of P_C8_T_8_ fibres in water droplets

The water phase was prepared by mixing aqueous stock solutions of **P**_**C8**_ (11.2 μL, 10 mM), MES buffer pH 6 (11.2 μL, 500 mM) and ThT (11.2 μL, 100 mM) in 78.2 μL of Milli-Q water. The oil phase contained 75 μL of Span 80 (80 mg mL^−1^ in dodecane) mixed with pure dodecane (1312 μL). The two solutions were mixed and shaken vigorously for 10 s at room temperature to form a w/o emulsion (w/o volume fraction, *φ*_W_ = 0.08). Fluorimetry measurements (ThT: ex = 450 nm; em = 482 nm) were performed under gentle stirring at 25 °C, triggering fibrillation by addition of neat octanal (**T**_**8**_, 20.5 μL) to a final concentration of 100 mM (oil phase). Seeded (auto-catalysis) experiments contain the pre-formed product (mol%) before addition of **T**_**8**_. Confined reaction and self-assembly were followed by HPLC (see ESI 2.7.)—**P**_**C2**_ was studied as control non-fibrillating precursor. For epifluorescence microscopic imaging (ThT: see above), 9 μL of the emulsion sample were added to a microscopy slide and fibrillation was triggered by addition of **T**_**8**_ (1 μL, 1.74 M in dodecane).

### Dye uptake and coalescence by droplets

Emulsions were prepared as indicated above, only now containing the corresponding fluorescent dye (10 µM final concentration in the water phase; stocks prepared in water) before fibrillation. Epifluorescence microscopic imaging was performed likewise (ThT, see above) (Rhodamine B/6G: ex = 540/25 nm, em = 605/55 nm) (Hoechst: ex = 375/28 nm, em = 460/60 nm). Relative dye uptake was calculated by the drop in external dye emission from the oil phase before and after fibrillation (Supplementary Fig. 9). Relative droplet coalescence was calculated by the relative increase in mean droplet size before and after fibrillation (Supplementary Fig. 10).

### Enzymatic cascade between droplet populations

Emulsions were prepared as indicated above, now including GOx (0.1 mg/mL)—tagged with DyLight 405 dye to aid visualisation—and Amplex Red (10 μM) to the water phase of one emulsion and HRP (10 U/mL) and glucose (50 mM) to the water phase of the second emulsion prior to emulsification. The two separate emulsions were sequentially added to a microscope slide in equal volumes and the reaction was initiated by addition of octanal (**T**_**8**_) (1 μL, 1.74 M in dodecane) and monitored by epifluorescence microscopic imaging (ThT, see above) (resorufin: ex = 540/25 nm, em = 605/55 nm) (GOx-DyLight 405: ex = 375/28 nm, em = 460/60 nm). For bulk fluorimetric experiments (Fig. 4d), untagged GOx was used instead, and the two emulsions (GOx and HRP) were mixed 1:1 (1.5 mL total) and heated to 25 °C under gentle stirring. The reaction and subsequent self-assembly was initiated by addition of 20.5 μL of neat octanal (**T**_**8**_) to a final concentration of 100 mM (oil phase). The communication between the two populations was monitored by resorufin emission (ex = 570 nm; em = 585 nm). The control without **T**_**8**_ was carried out by replacing the octanal used to initiate the reaction with an equivalent volume of pure dodecane. **P**_**C2**_ was studied as control non-fibrillating precursor.

## Supplementary information

Supplementary Information

Description of Additional Supplementary Files

Supplementary Movie 1

Supplementary Movie 2

Supplementary Movie 3

Supplementary Movie 4

Supplementary Movie 5

Supplementary Movie 6a

Supplementary Movie 6b

Supplementary Movie 7a

Supplementary Movie 7b

Supplementary Movie 8

Supplementary Movie 9

Supplementary Movie 10a

Supplementary Movie 10b

Supplementary Movie 11

## Data Availability

All data supporting the findings of this study is presented in the manuscript, Supplementary Information file and Supporting Movies. Source data is available from the corresponding author upon reasonable request.
