# Supplementary material for: Supramolecular fibrillation of peptide amphiphiles induces environmental responses in aqueous droplets
Source: Nat Commun. 2021 Nov 5;12:6421. doi: 10.1038/s41467-021-26681-2 (PMC8571317; doi:10.1038/s41467-021-26681-2)
Supplement: Supplementary file 1 — Supplementary Information [file 41467_2021_26681_MOESM1_ESM.pdf]

Supramolecular Fibrillation of Peptide Amphiphiles Induces Environmental Responses in Aqueous Droplets

*Richard Booth, Ignacio Insua, Sahnawaz Ahmed, Alicia Rioboo and Javier Montenegro**

Supplementary Information

1. Materials and instruments 3

2. Methods 3

2.1. Fluorescence measurements 3

2.2. Fluorescence microscopy 3

2.3. Circular dichroism experiments 3

2.4. Critical micellar and fibrillar concentration (CMC and CFC) 3

2.5. Co-assembly of precursor and product 4

2.6. HPLC kinetics in aqueous solution 4

2.7. HPLC kinetics in the water-in-oil emulsion 4

2.8. HPLC kinetics data analysis 5

2.9. Scanning-transmission electron microscopy (STEM) 5

2.10. Dynamic surface tension (DST) measurements 6

3. Synthesis and chemical characterisation 7

3.1. Methyl-8-bromooctanoate 7

3.2. Methyl-8-[Boc](aminooxy)]octanoate 8

3.3. 8-[Boc](aminooxy)]octanoic acid 11

3.4. $\mathbf{P_{C8}}$ – Synthesis and characterisation 14

3.5. $\mathbf{P_{C8}T_8}$ – Synthesis and characterisation 15

3.6. **P_{C2}** – Synthesis and characterisation 17

3.7. **$P_{C2}T_8$** – Synthesis and characterisation 18

4. Supplementary figures 20

5. Supplementary references 27

1. MATERIALS AND INSTRUMENTS

Reagents were acquired from Sigma-Aldrich, TCI, Iris Biotech or Fisher Scientific and were used without further purification. Purified water was obtained from Millipore Milli-Q integral 5 water purification system. Analytical HPLC was carried out using an Agilent 1260 Infinity II equipped with an Agilent SB-C18 column and connected to a 6120 Quadrupole LC-MS. HR-MS was acquired using a Bruker MicroTOF instrument. ^{1}H NMR and ^{13}C NMR spectra were acquired using a Varian 300 MHz spectrometer or a DRX 500 MHz spectrometer. Chemical shifts (δ) are reported in ppm relative to $CDCl_3$ (δ = 7.24 ppm for ^{1}H and 77.0 ppm for ^{13}C), DMSO-d_6 (δ = 2.50 ppm) and D_2O (δ = 4.79 ppm).

2. METHODS

2.1. Fluorescence measurements

Fluorescence measurements were carried out on a Horiba FluoroMax-3 fluorometer and were acquired with stirring at 25°C. Thioflavin T at 10 μM was used as the fluorescent probe for all experiments, using an excitation wavelength of 450 nm and an emission wavelength of 482 nm.

2.2. Fluorescence microscopy

Fluorescence microscopy was carried out using a Nikon Ti microscope equipped with an Andro Zyla sCMOS camera with images taken at either 10x or 60x magnification and using an FITC filter cube for Thioflavin T fluorescence (excitation: 480/30 nm, emission: 535/45 nm), a TRITC filter cube for resorufin and rhodamine B/6G fluorescence (excitation: 540/25 nm, emission: 605/55 nm) and a DAPI filter cube for Hoechst and DyLight-405 (excitation: 375/28 nm, emission: 460/60 nm).

2.3. Circular dichroism experiments

Circular dichroism spectra were acquired in a Jasco J-1100 CD spectrometer. Data was obtained at 25°C in a 2 mm light path quartz cuvette after subtraction of the solvent background signal.

2.4. Critical micellar and fibrillar concentration (CMC and CFC)

500 μL solutions of $\mathbf{P_{C8}T_8}$ from 2.5 to 80 μM were prepared in 50 mM MES buffer pH 6.0 in triplicate. 1 μL of a pyrene stock solution in tetrahydrofuran (1 mM) was added to each sample. Pyrene fluorescence emission was recorded (excitation: 334 nm, emission: 345-500 nm). Then, the I_3:I_1 ratio (Supplementary Fig. 3) was calculated for each spectrum by dividing the emission intensity at 383 nm (third emission band) by that at 372 nm (first emission band). The CMC was calculated as the intercept between the two linear

regimes found in the I_3:I_1 plot. High concentrations (up to 1 mM) of $\mathbf{P_{C8}T_8}$ and $\mathbf{P_{C8}}$ were studied likewise, only in one replicate due to the higher amounts of material needed for this concentration range. $\mathbf{P_{C8}T_8}$'s CFC was identified as the second I_3:I_1 transition found in this higher concentration regime, corresponding to the one-dimensional elongation of micelles into fibres, whereas $\mathbf{P_{C8}}$ did not display neither CMC nor CFC at concentrations as high as 1 mM.

2.5. Co-assembly of precursor and product

Samples containing $\mathbf{P_{C8}}$ (1 mM) and increasing amounts of doping $\mathbf{P_{C8}T_8}$ (0, 2, 5, 10 and 15 % mol/mol) in 50 mM MES buffer pH 6.0 were stained with 0.1% v/v of a ThT solution in water (5 mM). Dopant blanks were prepared likewise without $\mathbf{P_{C8}}$, only containing $\mathbf{P_{C8}T_8}$ in buffer with ThT at the same concentration. Fluorescence spectra were recorded for all samples (excitation: 430 nm, emission: 440-600 nm). The fluorescence spectrum of each dopant blank (*e.g.* $\mathbf{P_{C8}T_8}$ 5%) was subtracted from the corresponding doped samples (*e.g.* $\mathbf{P_{C8}}$ 1 mM + $\mathbf{P_{C8}T_8}$ 5%) to remove the contribution of the dopant in the mixture (Supplementary Fig. 4 *left*). The maximum emission intensity of these blank-subtracted spectra was normalized to that of pure $\mathbf{P_{C8}}$ (*i.e.* 0% doping) in Supplementary Fig. 4 *right*.

2.6. HPLC kinetics in aqueous solution

Kinetic experiments in aqueous solution were carried out by first preparing 300 µL of an aqueous solution containing peptide head $\mathbf{P_{C8}}$ (1 mM) and MES buffer (50 mM, pH 6). 50 µL of this solution was added to an aqueous solution of *O*-benzylhydroxylamine (10 µL, 6.5 mM) to be used as the t_0 time-point. The reaction was initiated by addition of octanal ($\mathbf{T_8}$) (5 µL, 250 mM in dodecane) and stirred for the duration of the reaction. The subsequent time-points were taken out at the required intervals in the same manner as t_0. The solutions of the individual time points were each washed with hexane (2x, 1 mL), the residual hexane was evaporated and the aqueous solution basified with ammonia (10 µL, 10% w/v) and MeCN (40 µL) was added to aid solubility of the sample before HPLC analysis (C18, H_2O/MeCN + 0.1% v/v TFA). The solutions were sonicated for 5 min, filtered and analyzed by LC-MS. The seeding experiments were carried as before but the aqueous solution was doped with the pre-formed $\mathbf{P_{C8}T_8}$ (5% mol/mol, 50 µM) and the reaction was initiated with 1 mM octanal.

2.7. HPLC kinetics in the water-in-oil emulsion

Typically, the water phase of the emulsion was prepared by mixing peptide head $\mathbf{P_{C8}}$ (52.5 µL, 10 mM), MES buffer (52.5 µL, 500 mM, pH 6) and 420 µL of Milli-Q water. The oil phase was prepared by adding 350 µL

of SPAN 80 (80 mg·mL^{-1}) dissolved in dodecane to 6125 µL of pure dodecane and the w/o emulsion was made by mixing the two phases together at an aqueous/oil volume fraction (φ_W) of 0.08. 1 mL of this solution was added to an aqueous solution of *O*-benzylhydroxylamine (10 µL, 6.5 mM) to be used as the t_0 time-point. The reaction was initiated by addition of octanal (**T_8**) (8.7 µL, 1.8 M) and rotated during the reaction to avoid sedimentation. The subsequent time-points were taken out at the required intervals in the same manner as t_0. The solutions of the individual time points were centrifuged and the oil layer removed. The subsequent solutions of the individual time-points were worked up and analysed in the same manner as the aqueous solutions. The competition experiments were carried out in the same manner but with the additional peptide head **P_{C2}** (1 mM final concentration) was added to the aqueous solution prior to emulsification. The seeding experiments were carried out by addition of the pre-formed peptide amphiphile, **$P_{C8}T_8$**, to the aqueous solution (5% mol/mol, 50 µM) prior to emulsification.

2.8. HPLC kinetics data analysis

The collected data was analysed by taking the ratio of the integrals of the starting material peak and the product peak and converting this number into relative peak area of peptide starting material remaining, expressed as a percentage. This was done to mitigate the effect of unknown levels of dilution due to the extensive work-up procedure. The HPLC signals of the starting materials and products were analysed and an experiment specific extinction coefficient was calculated, the ratios of the coefficients were used to correct for the different HPLC signals of the starting material and product where necessary. The reactions were completed in triplicate with an average taken.

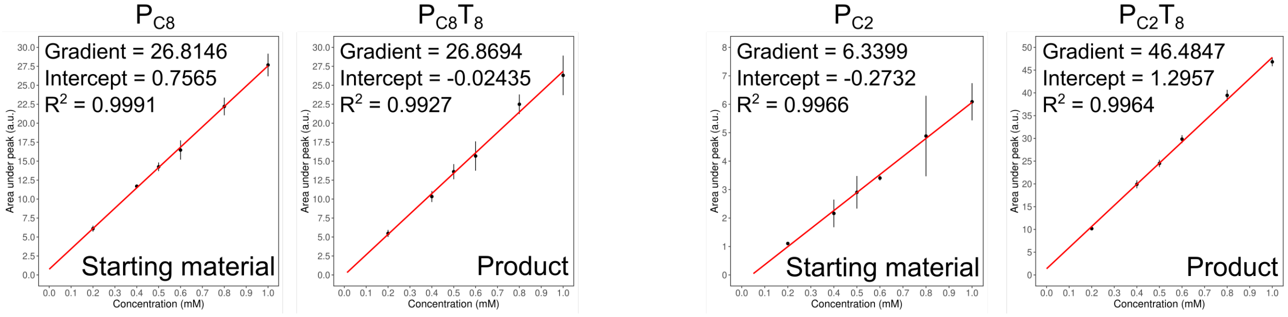

2.9. Scanning-transmission electron microscopy (STEM)

Scanning-transmission electron microscopy (STEM) images were acquired on a FESEM Ultra plus (Zeiss) operating at 20 kV. Liquid samples (MES buffer, pH 6.0) were cast on Cu grids (carbon type-B, 300 mesh, purchased from Ted Pella) and the excess sample was absorbed after 2 min of contact. The grid was then washed with MilliQ water (3x 40 µL) and stained with gadolinium (III) acetate.

2.10. Dynamic surface tension (DST) measurements

Dynamic surface tension (DST) measurements were carried out on a Krüss K100 using the maximum bubble pressure method,[1] with all measurements performed at 20°C and screening to the maximum surface age (*i.e.* inverse to bubble production rate) allowed by the instrument, $2.5 \cdot 10^7$ ms. Due to intense foaming, DST data from 1 mM $\mathbf{P_{C8}T_8}$ samples could only be acquired from surface age values of $7.5 \cdot 10^5$ ms onwards. Buffered $\mathbf{P_{C8}}$ (1 mM) samples represent a pre-fibrillated state, whereas increasing concentrations of buffered $\mathbf{P_{C8}T_8}$ simulate its progressive formation and fibrillation.

3. SYNTHESIS AND CHEMICAL CHARACTERISATION

3.1. Methyl-8-bromooctanoate

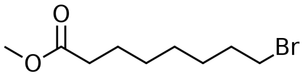

8-bromooctanoic acid (2 g, 8.96 mmol) was dissolved in 10 mL of methanol and stirred at room temperature for 10 min. H_2SO_4 (0.2 mL, 0.1 mL per gram of acid) was added and the solution was refluxed for 16 h. Methanol was removed under reduced pressure and afterwards the resultant solution was neutralized by addition of 10 mL of saturated $NaHCO_3$. The product was extracted from the aqueous solution by addition of DCM (3 x 2 mL). The organic phase was dried with anhydrous $MgSO_4$ and then removed under reduced pressure. The desired product was obtained as a light-yellow liquid (2.01 g, 95%).

NMR spectra: Compound already characterized[2] and commercially available.

3.2. Methyl-8-[Boc](aminooxy)]octanoate

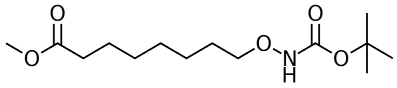

Methyl 8-bromooctanoate (2 g, 8.44 mmol) was dissolved in anhydrous MeCN (40 mL), was kept under argon and stirred for 10 min at room temperature. *Tert*-butyl-*N*-hydroxycarbamate (1.13 g, 8.44 mmol) and 1,8-diazabicyclo(5.4.0)undec-7-ene (3.78 mL, 25.32 mmol) were added and the solution was refluxed under argon for 16 h. The solution was neutralized by addition of saturated NH_4Cl (40 mL) and the subsequent solution was concentrated under reduced pressure. Once the MeCN was eliminated, the resulting aqueous solution was extracted by addition of DCM (3 x 15 mL) to yield a yellow liquid which was purified by column chromatography (5:1 hexane/ethyl acetate) affording methyl-8-[Boc](aminooxy)]octanoate as a colourless liquid (500 mg, 20 %).

^{1}H NMR ($CDCl_3$, 500 MHz): δ (ppm) 7.11 (s, 1H, NH), 3.83 (t, *J*=6.6 Hz, 2H, OCH_2), 3.66 (s, 3H, OCH_3), 2.29 (t, *J*=7.5 Hz, 2H, $COCH_2$), 1.65-1.56 (m, 4H, 2 x CH_2), 1.47 (s, 9H, 3 x CH_3), 1.39-1.28 (m, 6H, 3 x CH_2).

^{13}C NMR ($CDCl_3$, 125 MHz): δ (ppm) 174.38 (C=O), 157.05 (C=O), 81.68 (*C*O(CH_3)$_3$), 77.17 (CH_2O), 51.58 (CH_3), 34.17 (CH_2), 29.15 (CH_2), 29.11 (CH_2), 28.37 (3 x CH_3), 28.07 (CH_2), 25.82 (CH_2), 24.97 (CH_2).

HRMS (ESI, m/z): Calculated for $C_{14}H_{27}NNaO_5$: 312.1781; found: 312.1782 ($[M+Na]^+$).

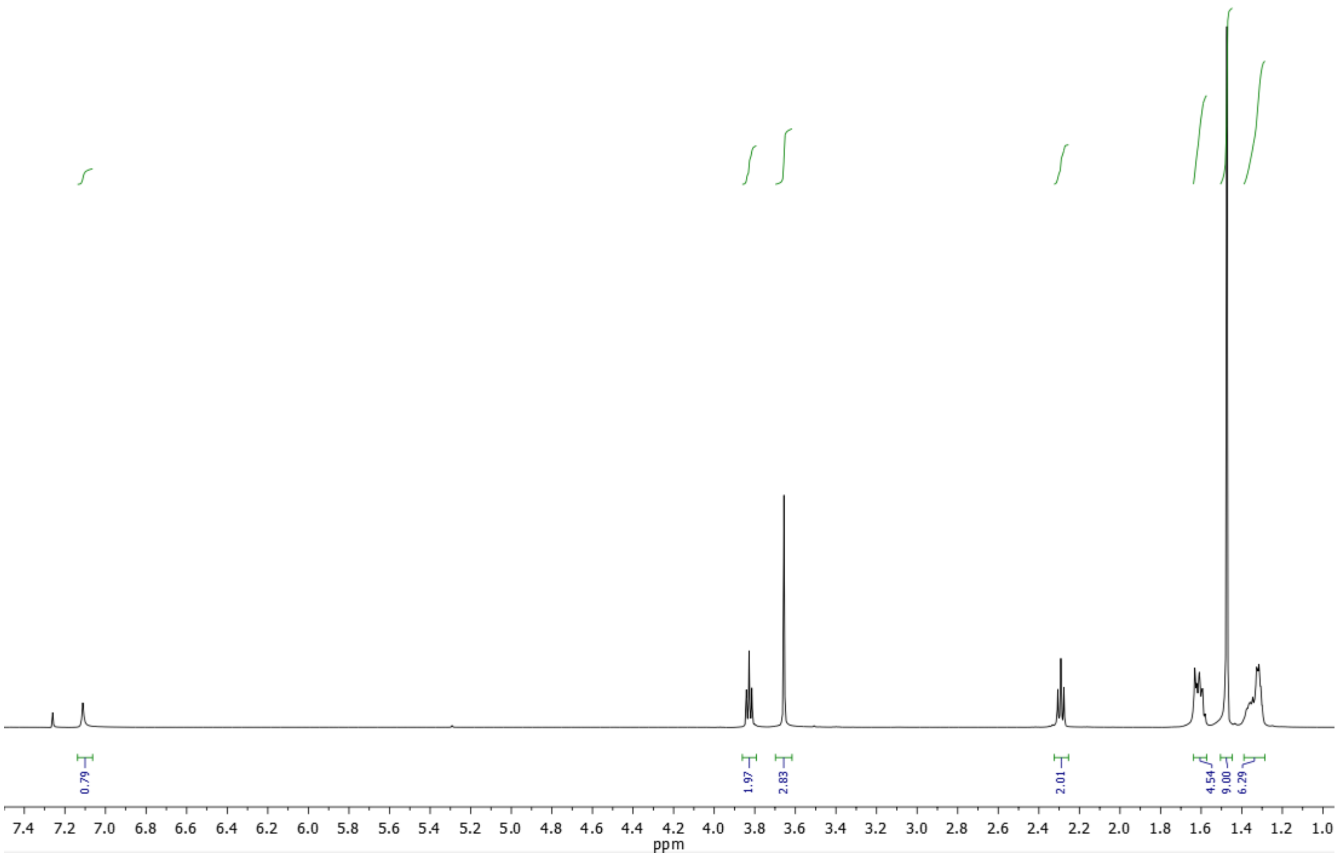

^{1}H NMR ($CDCl_3$, 500 MHz) of methyl-8-[Boc](aminooxy)]octanoate

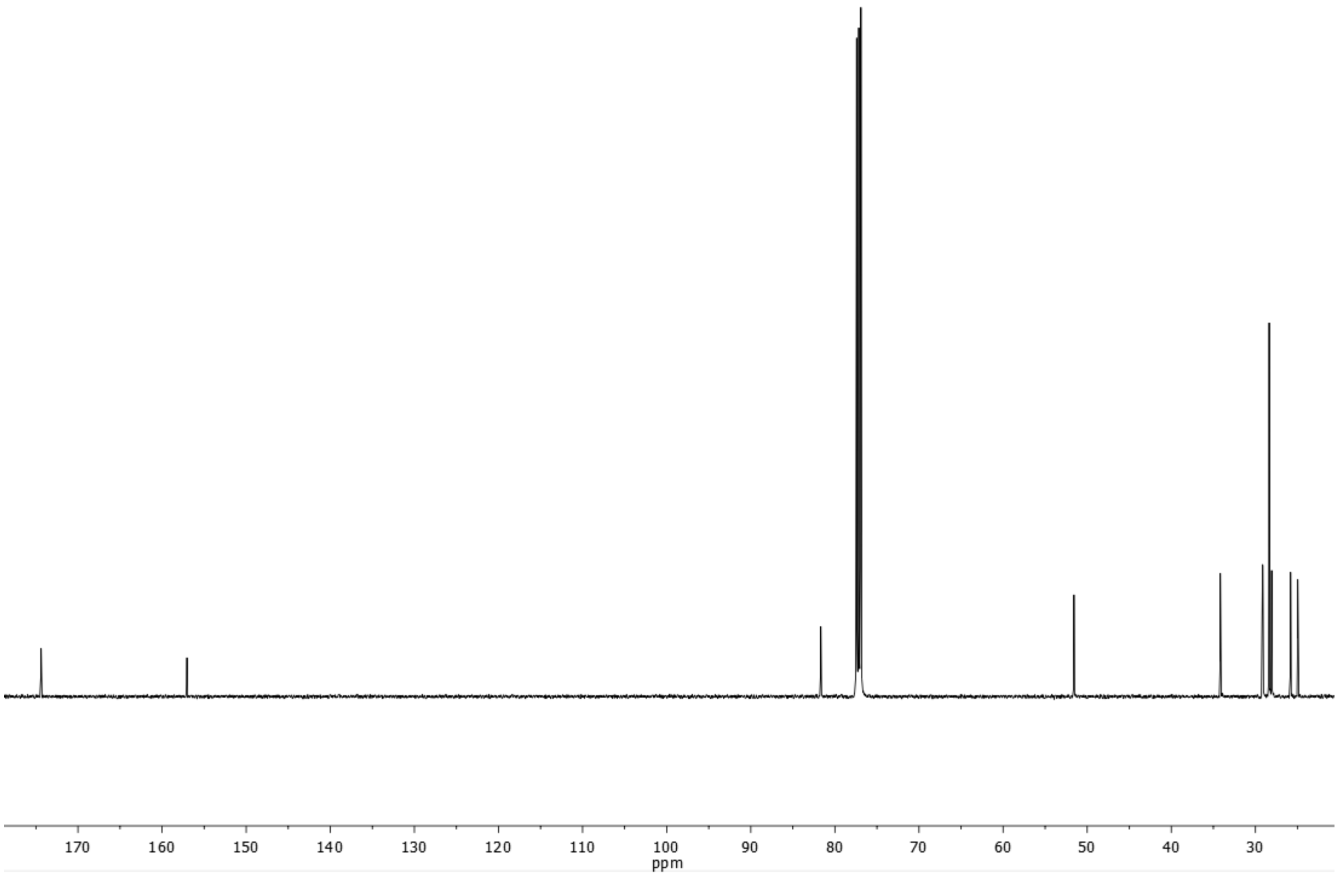

^{13}C NMR ($CDCl_3$, 125 MHz) of methyl-8-[Boc](aminooxy)]octanoate

3.3. 8-[Boc](aminooxy)]octanoic acid

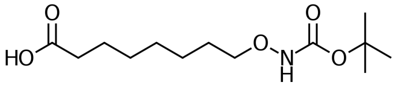

Methyl-8-[Boc](aminooxy)]octanoate (350 mg, 1.21 mmol) was dissolved in 10 mL of methanol. To this solution, a solution of NaOH 2N was added until a white turbidity has been observed. The solution was stirred at 40°C for 3 h. The methanol was removed under reduced pressure and the subsequent aqueous phase was acidified to pH 3.5 by addition of HCl (10%). The product was extracted from the aqueous solution by addition of DCM (3 x 10 mL). The organic phase was dried with anhydrous $MgSO_4$ and removed under reduced pressure to obtain the product as a colourless liquid (295 mg, 89%).

^{1}H NMR ($CDCl_3$, 500 MHz): δ (ppm) 7.29 (s, 1H NH), 3.83 (t, *J*=6.6 Hz, 2H, OCH_2), 2.34 (t, *J*=7.5 Hz, 2H, $COCH_2$), 1.68-1.58 (m, 4H, 2 x CH_2), 1.48 (s, 9H, 3 x CH_3), 1.41-1.30 (m, 6H, 3 x CH_2).

^{13}C NMR ($CDCl_3$, 125 MHz): δ (ppm) 179.42 (C=O), 157.26 (C=O), 81.84 (*C*O(CH_3)$_3$), 77.18 (CH_2O), 34.05 (CH_2), 29.12 (CH_2), 29.03 (CH_2), 28.38 (3 x CH_3), 28.06 (CH_2), 25.80 (CH_2), 24.71 (CH_2).

HRMS (ESI, m/z): Calculated for $C_{13}H_{25}NNaO_5$: 298.1625; found: 298.1625 ($[M+Na]^+$).

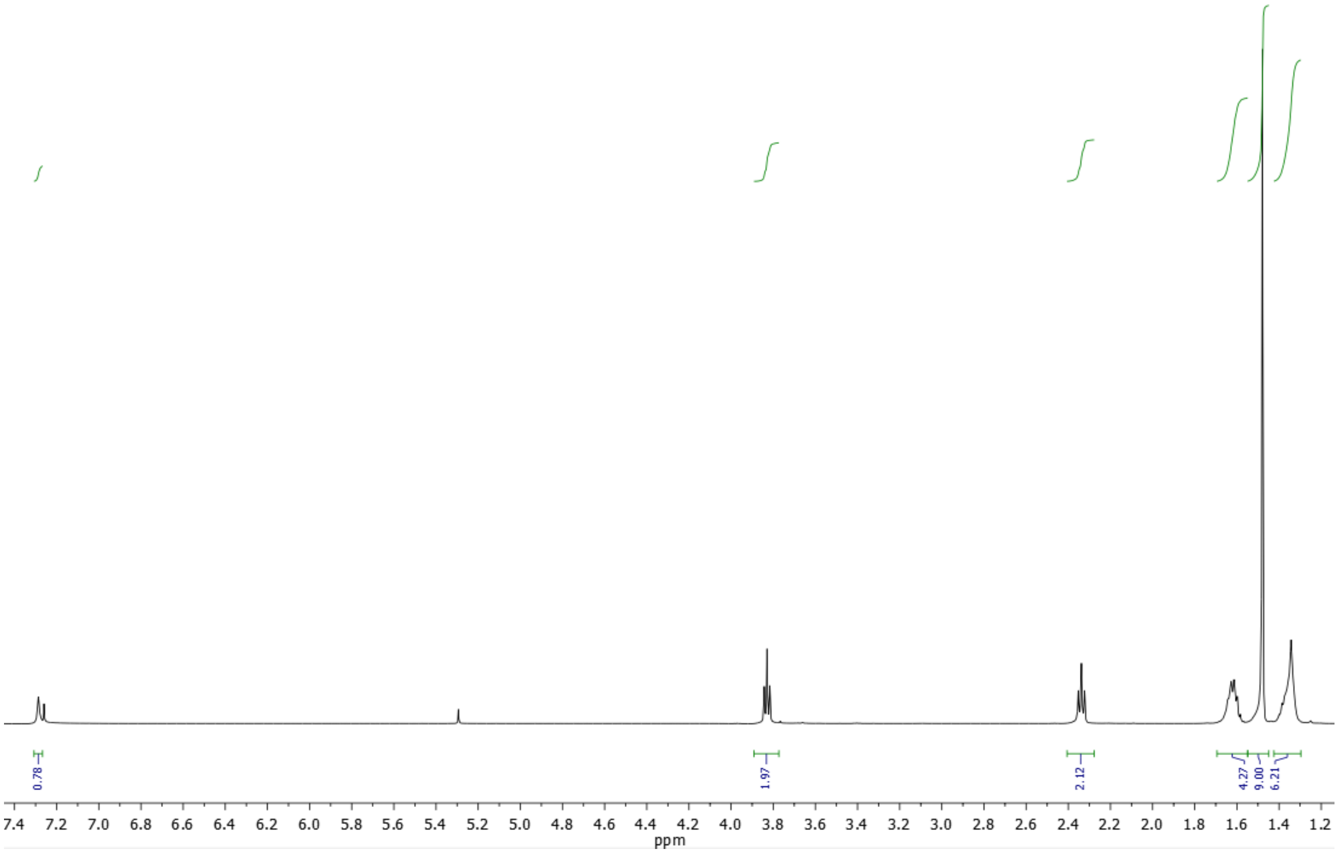

¹H NMR ($CDCl_3$, 500 MHz) of 8-[Boc](aminooxy)]octanoic acid

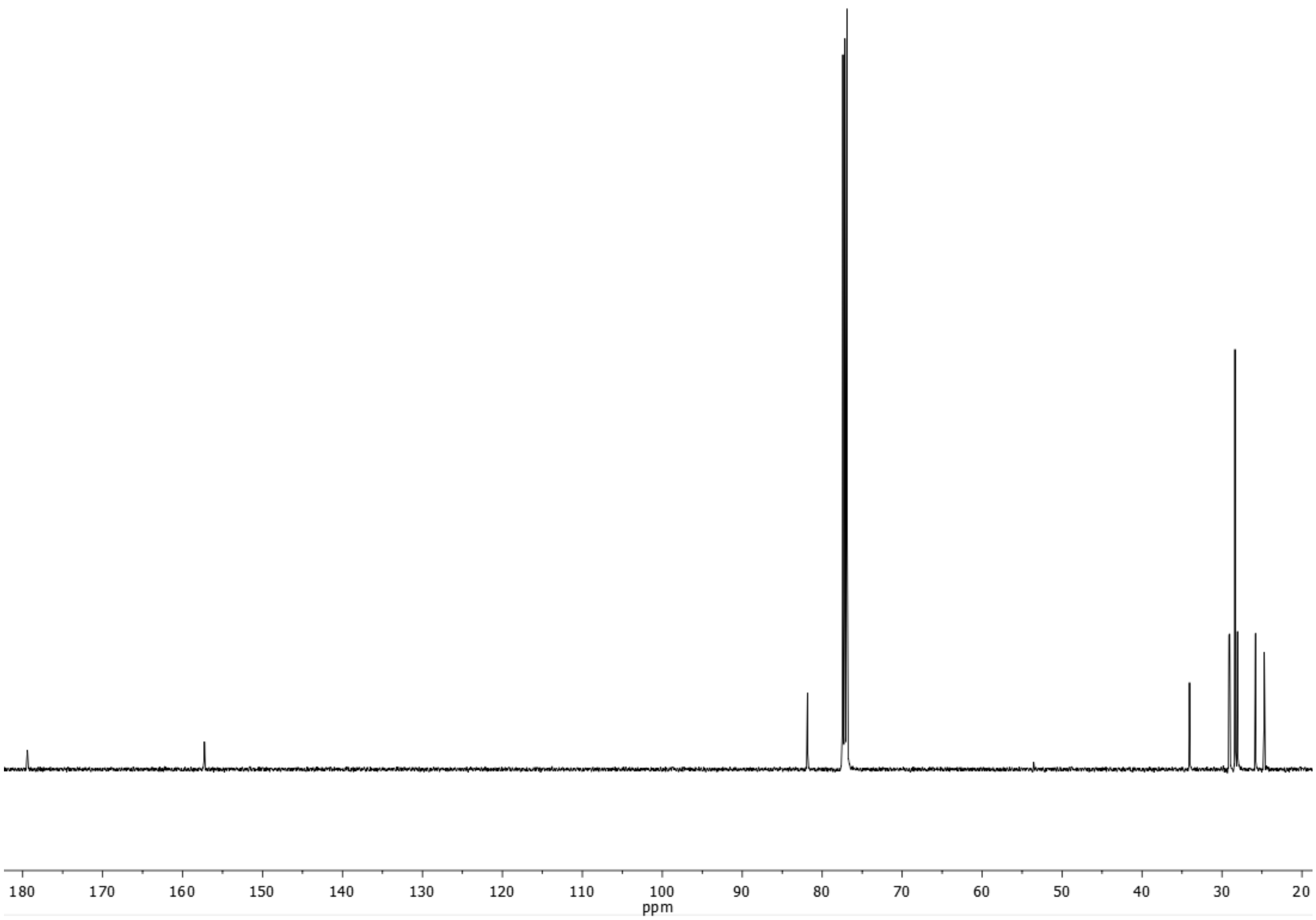

^{13}C NMR ($CDCl_3$, 125 MHz) of 8-[Boc](aminooxy)]octanoic acid

3.4. P_{C8} – Synthesis and characterisation

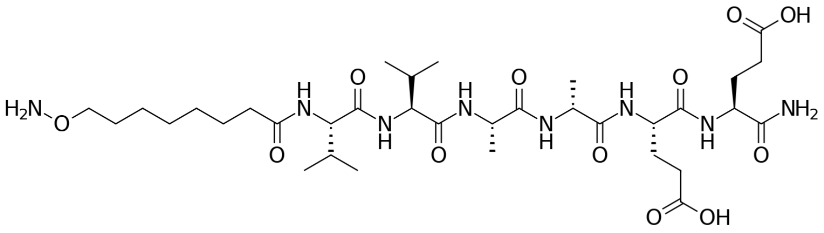

P_{C8} was synthesized following the peptide synthesis general procedure, starting from a 0.1 mmol scale of Rink Amide resin. The desired product has been obtained as a white powder (29.4 mg, 38%).

^{1}H NMR (D_2O, 300 MHz): δ (ppm) 4.39-4.20 (m, 4H, Hα), 4.14-3.99 (m, 4H, Hα x2 + aliphatic OCH_2), 2.53-2.41 (m, 4H, Asp -CH_2- x2), 2.34-2.22 (m, 2H, aliphatic $COCH_2$), 2.20-1.91 (m, 6H, Asp -CH_2- x2 + Val -CH- x2), 1.72-1.49 (m, 4H, aliphatic CH_2 x2), 1.43-1.22 (m, 12H, Ala -CH_3 x2 + aliphatic CH_2 x3), 0.98-0.84 (m, 12H, Val -CH-$(CH_3)_2$ x2).

HRMS (ESI, m/z): Calculated for $C_{34}H_{61}N_8O_{12}$: 773.4403; found: 773.4408 ($[M+H]^+$).

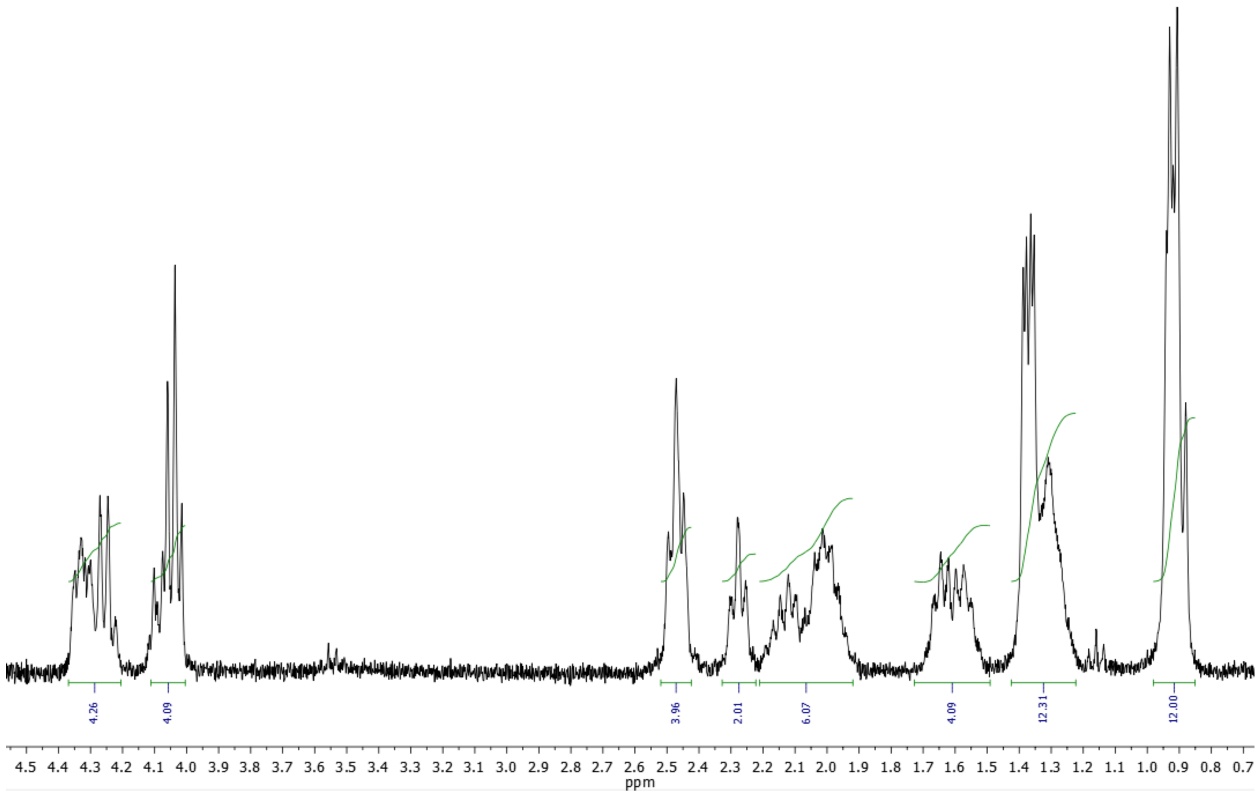

^{1}H NMR (D_2O, 300 MHz) of **P_{C8}**

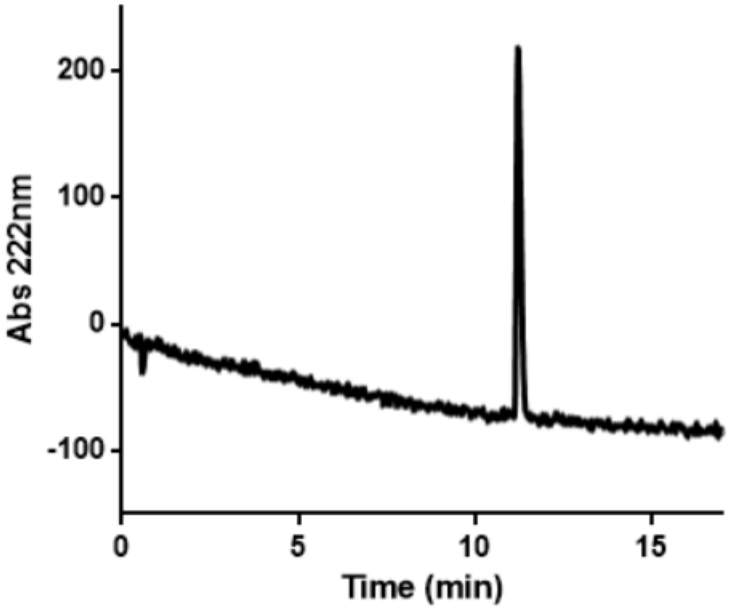

HPLC of $\mathbf{P_{C8}}$ obtained in a C18 column with eluent CH_3CN-0.1%TFA:H_2O-0.1%TFA (0:100) to CH_3CN-0.1%TFA:H_2O-0.1%TFA (50:50) in 15 min, with 2 initial minutes in isocratic at CH_3CN-0.1%TFA:H_2O-0.1%TFA (0:100).

3.5. $\mathbf{P_{C8}T_8}$ – Synthesis and characterisation

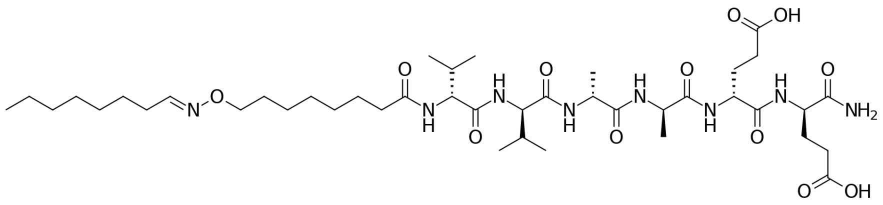

$\mathbf{P_{C8}T_8}$ was synthesized following the peptide amphiphile general synthesis procedure, starting from 5 mg of $\mathbf{P_{C8}}$. The desired product has been obtained as a white powder (5.5 mg, 96%).

^{1}H NMR (DMSO-d_6, 300 MHz): δ (ppm) 8.18 (d, *J*=7.2Hz, 1H, CONH), 8.04 (d, *J*=6.8Hz, 2H, CONH x2), 7.94-7.78 (m, 2H, CONH x2), 7.76 (d, *J*=7.6Hz, 1H, CONH), 7.37 (t, *J*=6.2Hz, 0.5H, NCH oxime *trans*), 7.21 (s, 1H, NH_2 C terminus), 7.06 (s, 1H, NH_2 C terminus), 6.69 (t, *J*=5.4Hz, 0.5H, NCH oxime *cis*), 4.36-4.03 (m, 6H, Hα x6), 4.01-3.83 (m, 2H, aliphatic OCH_2), 2.30-2.04 (m, 8H, Asp -CH_2- x2 + aliphatic $COCH_2$ + aliphatic CH_2CHNO), 2.04-1.85 (m, 4H, Asp -CH_2- x2), 1.85-1.66 (m, 2H, Val -CH- x2), 1.62-1.36 (m, 6H, aliphatic CH_2 x3), 1.36-1.12 (m, 20H, Ala -CH_3 x2 + aliphatic CH_2 x7), 0.87-0.77 (m, 15H, Val -CH-(CH_3)$_2$ x2 + aliphatic CH_3).

HRMS (ESI, m/z): Calculated for $C_{42}H_{75}N_8O_{12}$: 883.5499; found: 883.5516 ($[M+H]^+$).

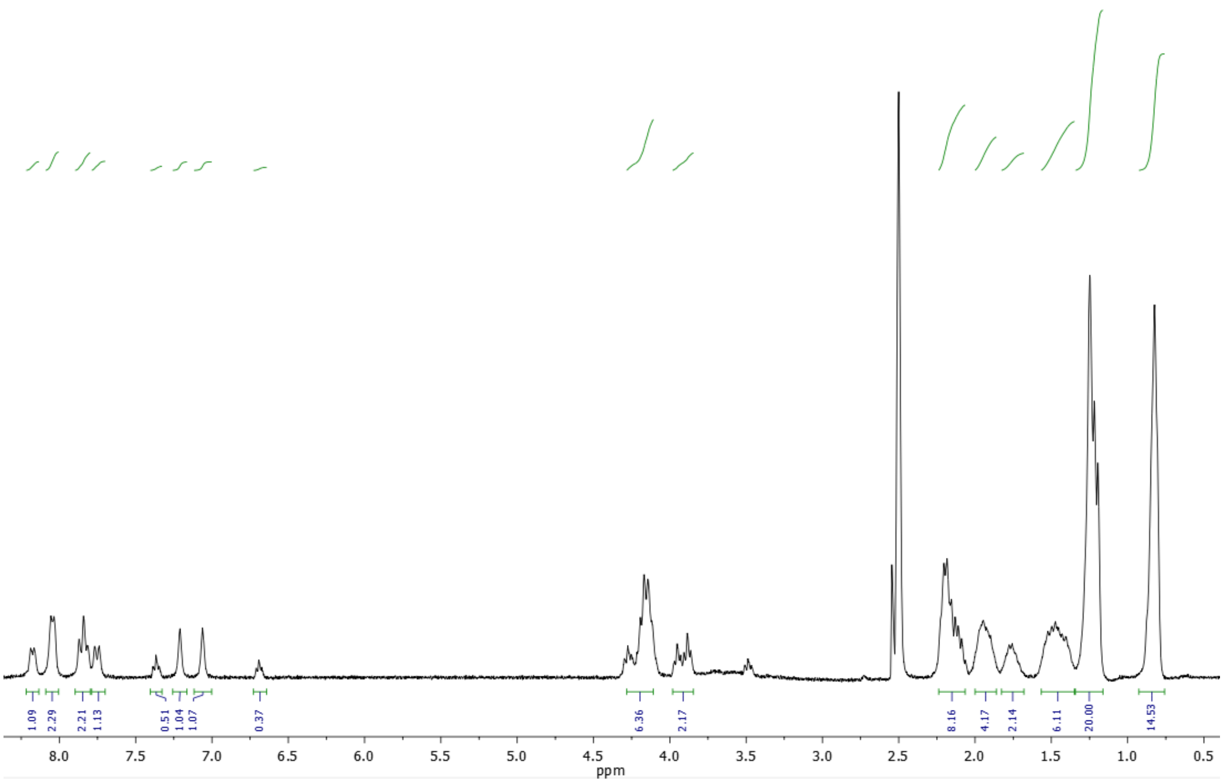

^{1}H NMR (DMSO-*d_6*, 300 MHz) of **$P_{C8}T_8$**

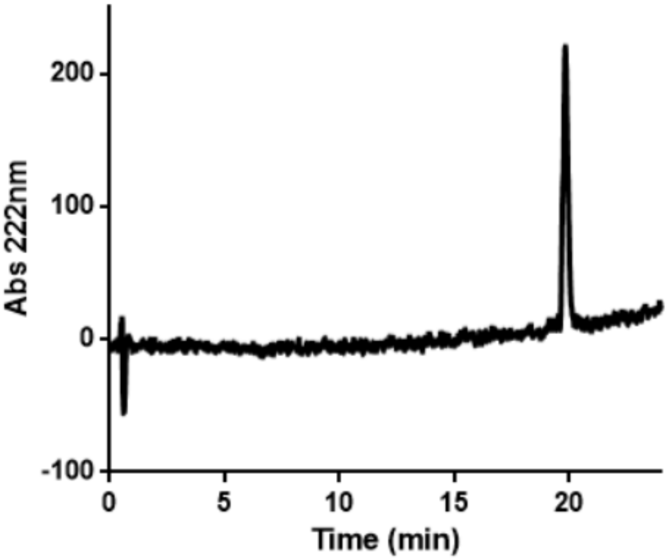

HPLC of **$P_{C8}T_8$** obtained in a C18 column with eluent CH_3CN-0.1%TFA:H_2O-0.1%TFA (0:100) to CH_3CN-0.1%TFA:H_2O-0.1%TFA (75:25) in 22 min, with 2 initial minutes in isocratic at CH_3CN-0.1%TFA:H_2O-0.1%TFA (0:100).

3.6. P_{C2} – Synthesis and characterisation

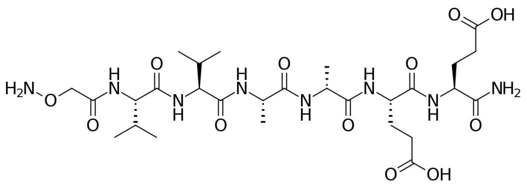

P_{C2} was synthesized following the peptide synthesis general procedure, starting from a 0.1 mmol scale of Rink Amide resin. The desired product has been obtained as a white powder (21.1 mg, 31%).

^{1}H NMR (D_2O, 300 MHz): δ (ppm) 4.33-4.21 (m, 4H, Hα x4), 4.25 (s, 2H, aliphatic CH_2), 4.18 (d, *J*=8.0Hz, 1H, Val Hα), 4.12 (d, *J*=8.2Hz, 1H, Val Hα), 2.36-2.23 (m, 4H, Asp -CH_2- x2), 2.14-1.86 (m, 6H, Asp -CH_2- x2 + Val -CH- x2), 1.39 (d, *J*=7.2Hz, 3H, Ala -CH_3), 1.39 (d, *J*=7.2Hz, 3H, Ala -CH_3), 1.01-0.85 (m, 12H, Val -CH-(CH_3)$_2$ x2).

HRMS (ESI, m/z): Calculated for $C_{28}H_{49}N_8O_{12}$: 689.3464; found: 689.3466 ($[M+H]^+$).

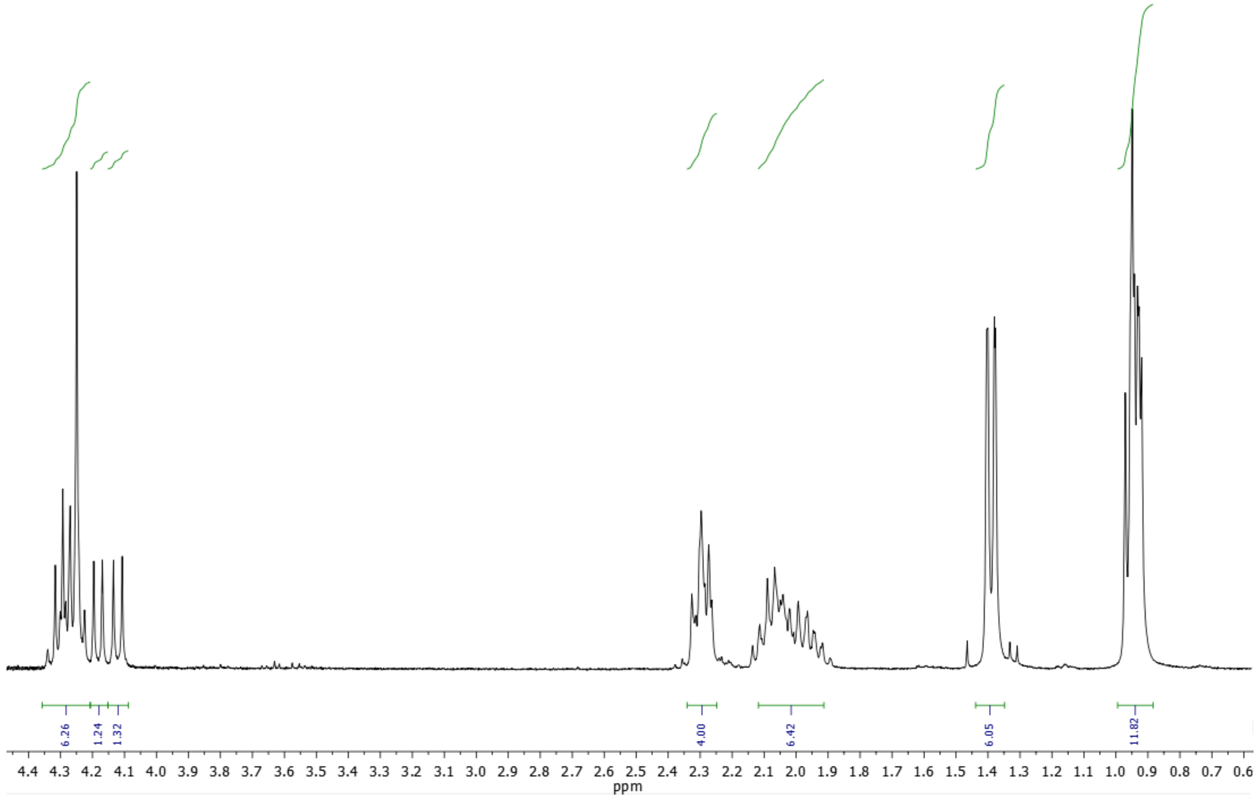

^{1}H NMR (D_2O, 300 MHz) of **P_{C2}**

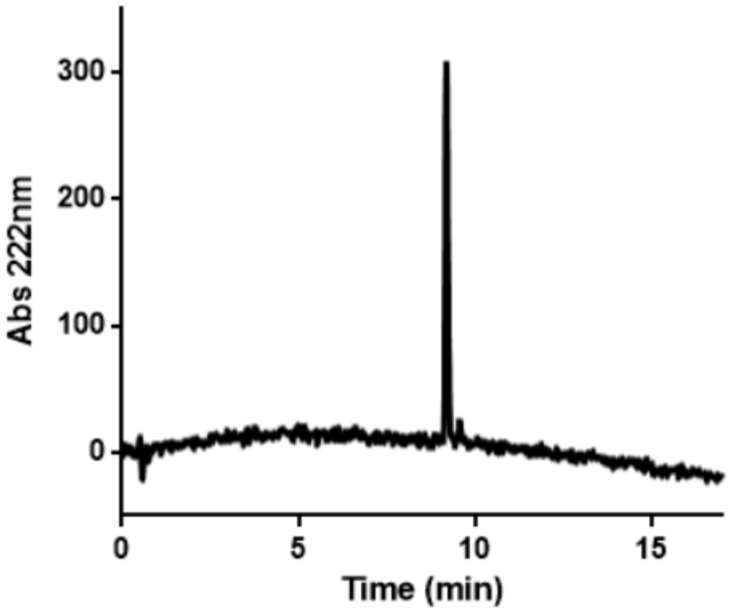

HPLC of $\mathbf{P_{C2}}$ obtained in a C18 column with eluent CH_3CN-0.1%TFA:H_2O-0.1%TFA (0:100) to CH_3CN-0.1%TFA:H_2O-0.1%TFA (50:50) in 15 min, with 2 initial minutes in isocratic at CH_3CN-0.1%TFA:H_2O-0.1%TFA (0:100).

3.7. $\mathbf{P_{C2}T_8}$ – Synthesis and characterisation

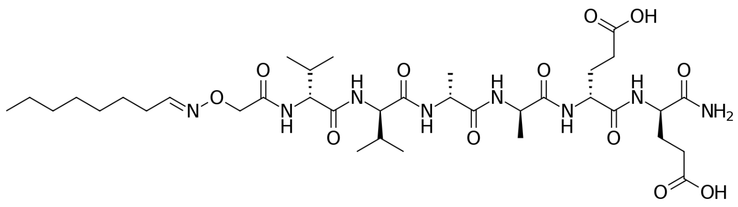

$\mathbf{P_{C2}T_8}$ was synthesized following the peptide amphiphile general synthesis procedure, starting from 5 mg of $\mathbf{P_{C8}}$. The desired product has been obtained as a white powder (5.1 mg, 91%).

^{1}H NMR (DMSO-d_6, 300 MHz): δ (ppm) 8.11-7.90 (m, 3H, CONH x3), 7.82 (d, *J*=7.8Hz, 1H, CONH), 7.61-7.54 (m, 1H, CONH), 7.41 (d, *J*=8.8Hz, 1H, CONH), 7.35-7.30 (m, 0.5H, NCH oxime *trans*), 7.26 (s, 1H, NH_2 C terminus), 7.07 (s, 1H, NH_2 C terminus), 6.88-6.82 (m, 0.5H, NCH oxime *cis*), 4.49-4.08 (m, 8H, Hα x6 + aliphatic OCH_2), 2.38-2.05 (m, 8H, Asp -CH_2- x2 + Val -CH- x2 + aliphatic CH_2CHNO), 2.05-1.84 (m, 4H, Asp -CH_2- x2), 1.83-1.64 (m, 2H, aliphatic CH_2), 1.51-1.34 (m, 2H, aliphatic CH_2), 1.35-1.12 (m, 12H, Ala -CH_3 x2 + aliphatic CH_2 x3), 0.94-0.71 (m, 15H, Val -CH-(CH_3)$_2$ x2 + aliphatic CH_3).

HRMS (ESI, m/z): Calculated for $C_{36}H_{63}N_8O_{12}$: 799.4560; found: 799.4569 ([M+H]$^+$).

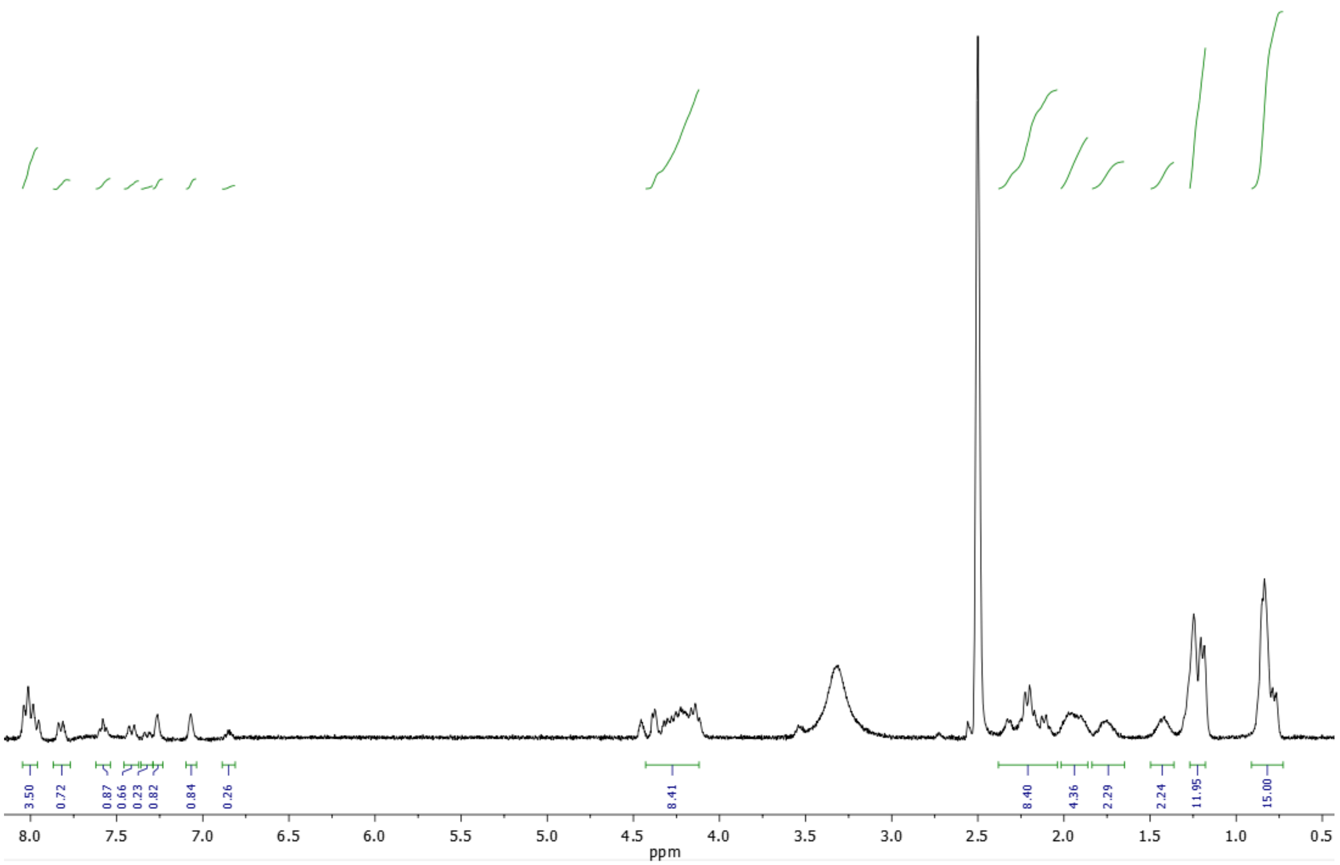

^{1}H NMR (DMSO-d_6, 300 MHz) of **$P_{C2}T_8$**

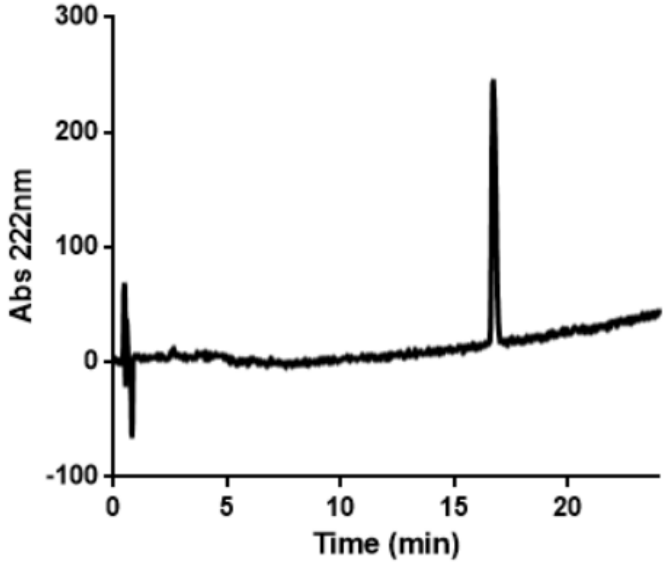

HPLC of **$P_{C2}T_8$** obtained in a C18 column with eluent CH_3CN-0.1%TFA:H_2O-0.1%TFA (0:100) to CH_3CN-0.1%TFA:H_2O-0.1%TFA (75:25) in 22 min, with 2 initial minutes in isocratic at CH_3CN-0.1%TFA:H_2O-0.1%TFA (0:100).

4. SUPPLEMENTARY FIGURES

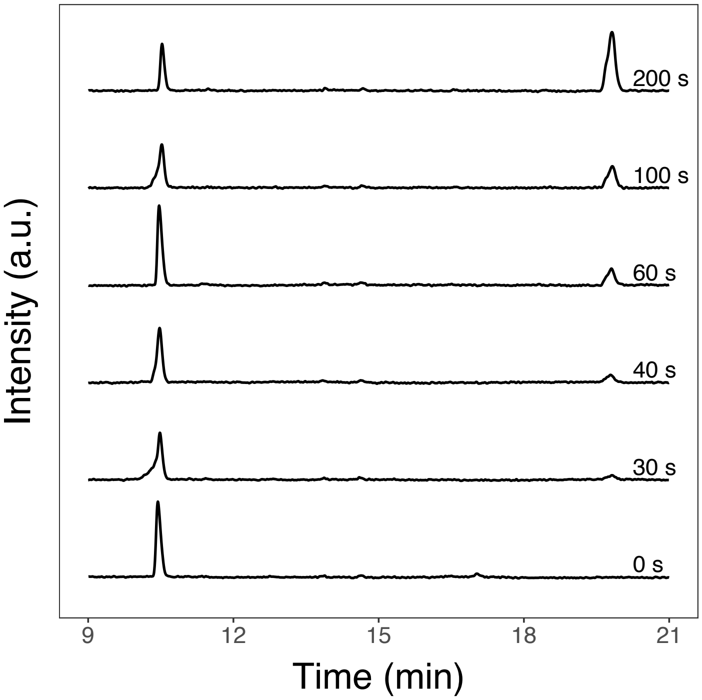

Supplementary Figure 1. HPLC chromatograms (222 nm) showing the conversion of the peptide head (**P_{C8}**, R_t = 10.5 min) to peptide amphiphile (**$P_{C8}T_8$**, R_t = 20 min) over time after the addition of octanal (**T_8**).

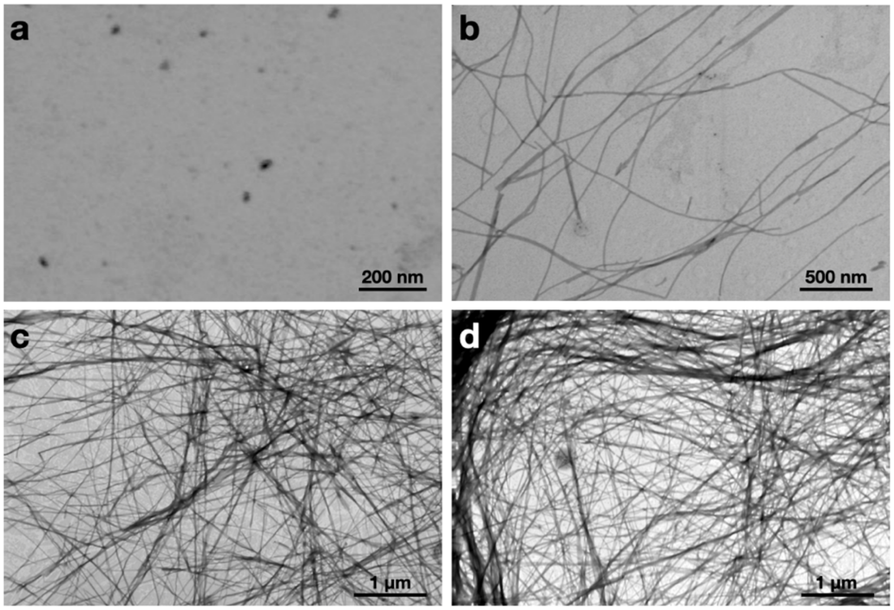

Supplementary Figure 2. STEM images of **P_{C8}** precursor (250 μM, **a**) and **$P_{C8}T_8$** fibres (increasing concentrations up to 1 mM, **b**-**d**).

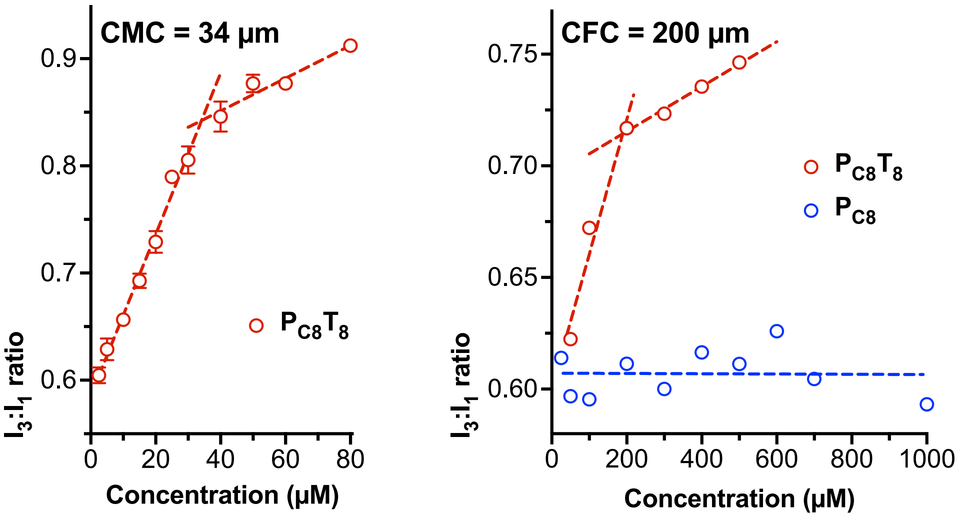

Supplementary Figure 3. Relative pyrene emission (I_3:I_1 ratio; see methods above) in presence of $\mathbf{P_{C8}T_8}$ and $\mathbf{P_{C8}}$ at low (left, *n*=3, mean±SD) and high concentrations (right, *n*=1). The calculated critical micellar and fibrillar concentrations, CMC and CFC respectively, are indicated above the intercept of the corresponding linear regressions.

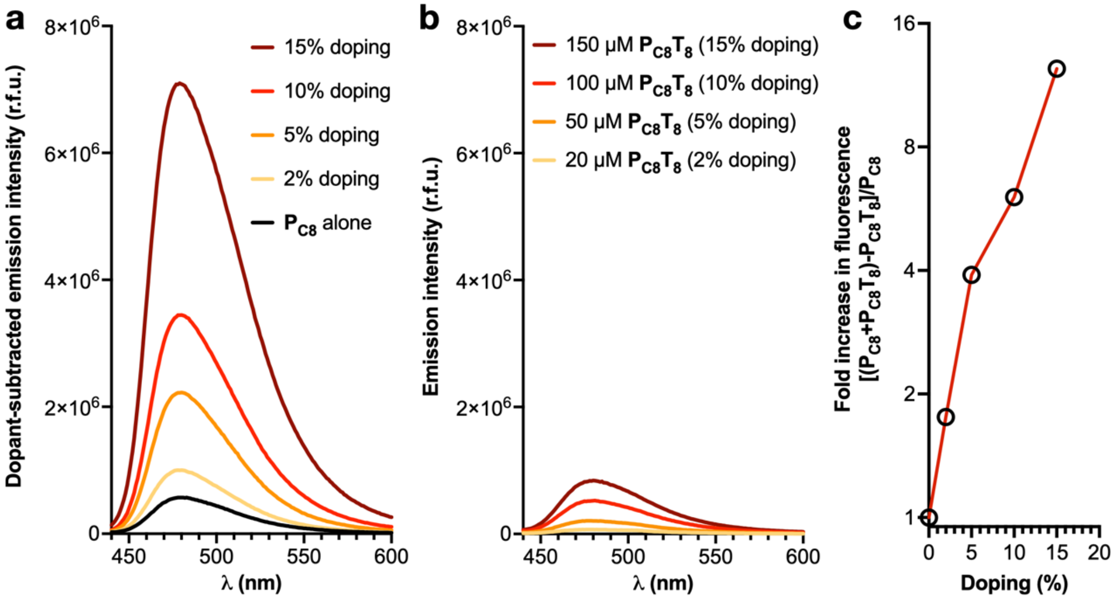

Supplementary Figure 4. (**a**) ThT emission spectra in presence of $\mathbf{P_{C8}}$ (1 mM) doped with increasing amounts of its reaction product $\mathbf{P_{C8}T_8}$ (0-15% mol/mol). The contribution of the doping $\mathbf{P_{C8}T_8}$ has been subtracted (see methods). (**b**) ThT emission in presence of the dopants alone as controls. (**c**) Fold increase in dopant-corrected emission -plot (**a**)- relative to doping % (mol/mol).

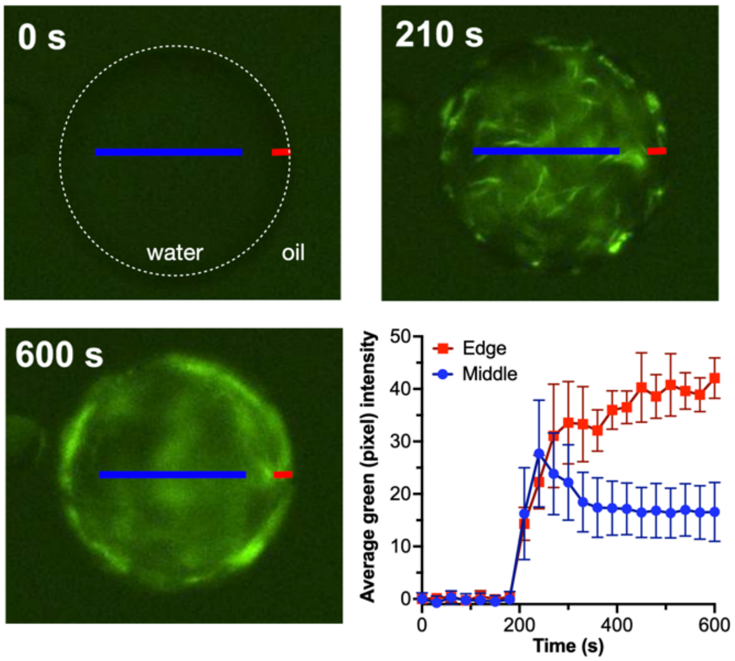

Supplementary Figure 5. Representative time-lapse epifluorescence micrographs of a single droplet loaded with $\mathbf{P_{C8}}$ undergoing fibrillation with $\mathbf{T_8}$ (see Fig. 2e). Fibres stained with ThT (green emission). The plot represents the average pixel intensity (green emission) of the blue (middle) and red (edge) cross-sections.

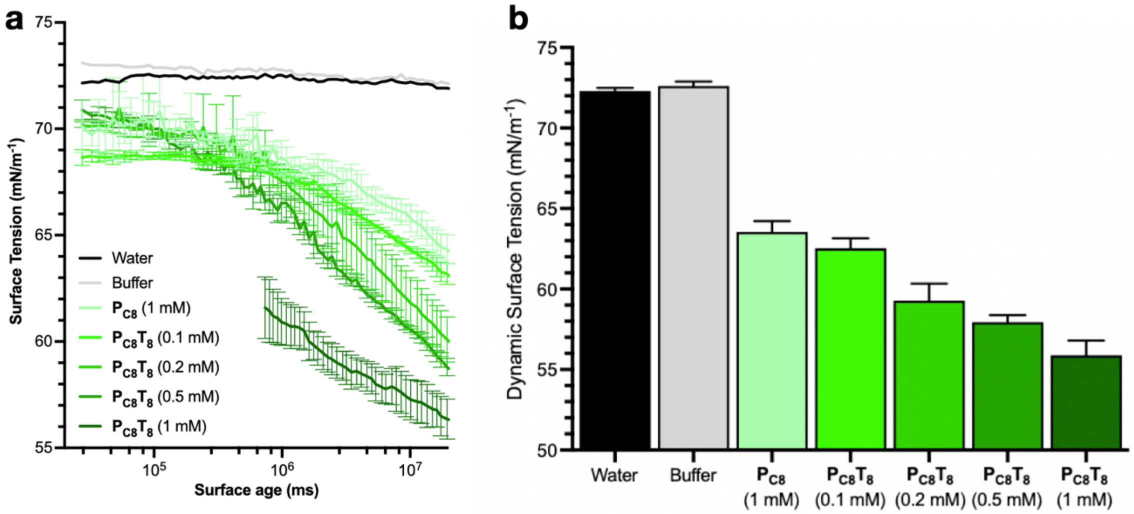

Supplementary Figure 6. (**a**) Dynamic surface tension (DST) profiles obtained from the maximum bubble pressure method representing surface tension of different solutions versus slower bubble production rates (*i.e.* increasing surface ages).[1] All $\mathbf{P_{C8}}$ and $\mathbf{P_{C8}T_8}$ samples were prepared in buffer (50 mM MES pH 6.0); *n*=3 (mean±SD). (**b**) DST values at the highest surface age measured ($2.5 \cdot 10^7$ ms). For water and plain buffer (with static surface tensions) the value plotted is the average of all surface ages.

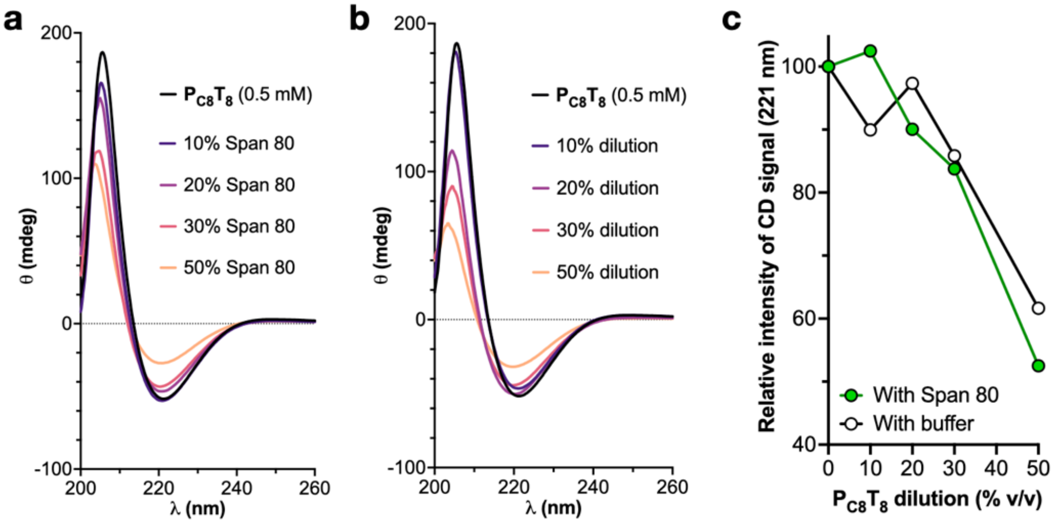

Supplementary Figure 7. Circular dichroism spectra of $\mathbf{P_{C8}T_8}$ fibres (0.5 mM) in buffer (50 mM MES, pH 6.0) diluted with a 0.5 mM stock of the surfactant Span 80 (**a**) or buffer (**b**). Percentages indicate %vol/vol. Being $\mathbf{P_{C8}T_8}$ and Span 80 stocks at the same concentration, %vol/vol = %mol/mol. (**c**) Relative intensity of the 221 nm band versus dilution with Span 80 (green) or buffer (white). Pure $\mathbf{P_{C8}T_8}$ 0.5 mM = 100% signal.

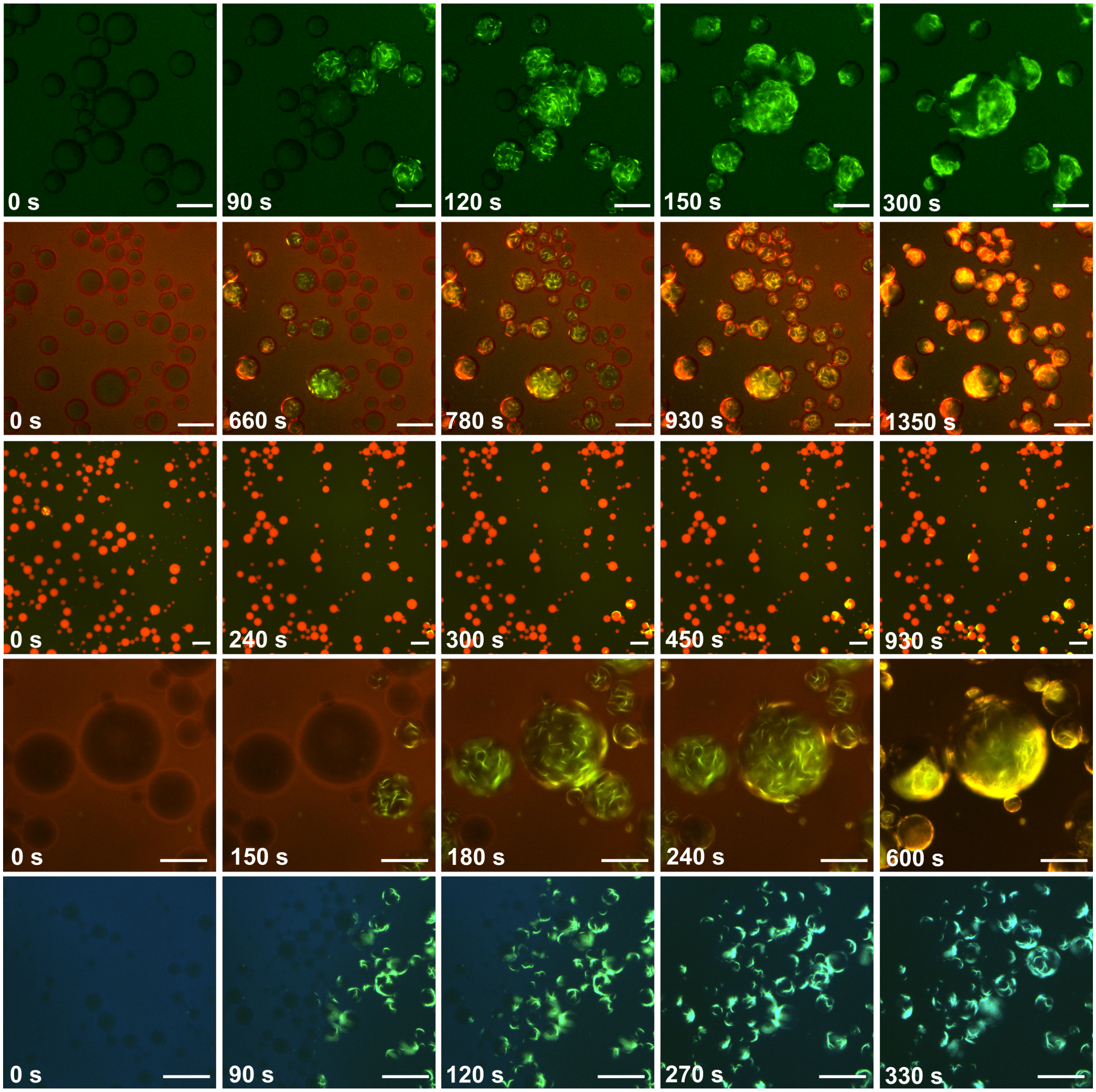

Supplementary Figure 8. Fluorescence microscopy images showing the coalescence of droplets without any external dye (top row), not showing any coalescence in the presence of the zwitterionic Rhodamine B (2nd row), or any coalescence in the presence of the anionic Rhodamine-based TAMRA (3rd row), while exhibiting enhanced coalescence in the presence of the cationic dyes Rhodamine 6G (4th row) and Hoechst 33342 (5th row). Times are since the addition of octanal (**T₈**). All scale bars = 100 μm.

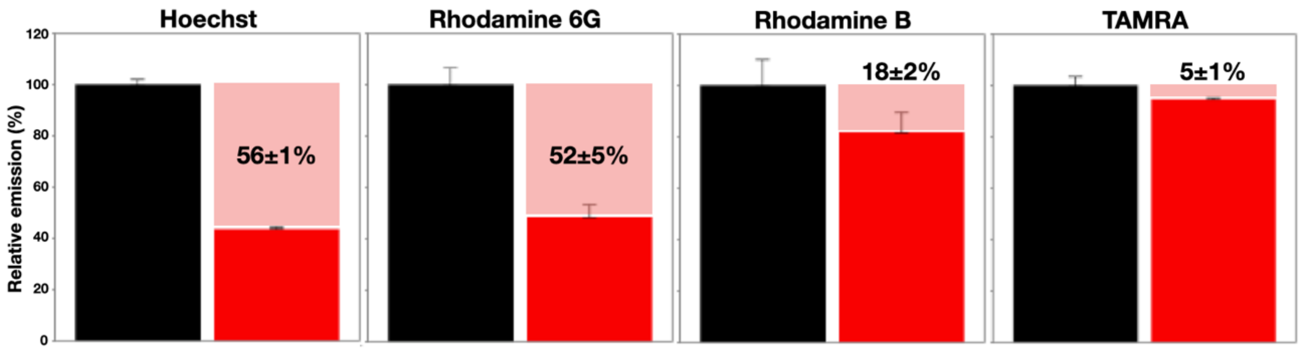

Supplementary Figure 9. Average emission of each dye in the oil phase of the emulsion before (black) and after (red) fibrillation. Dye emission before fibrillation is normalised to 100%. The difference in dye emission before and after fibrillation is represented in light pink and labelled numerically, representing the relative amount of dye taken up by droplets; *n*=5(mean±SD).

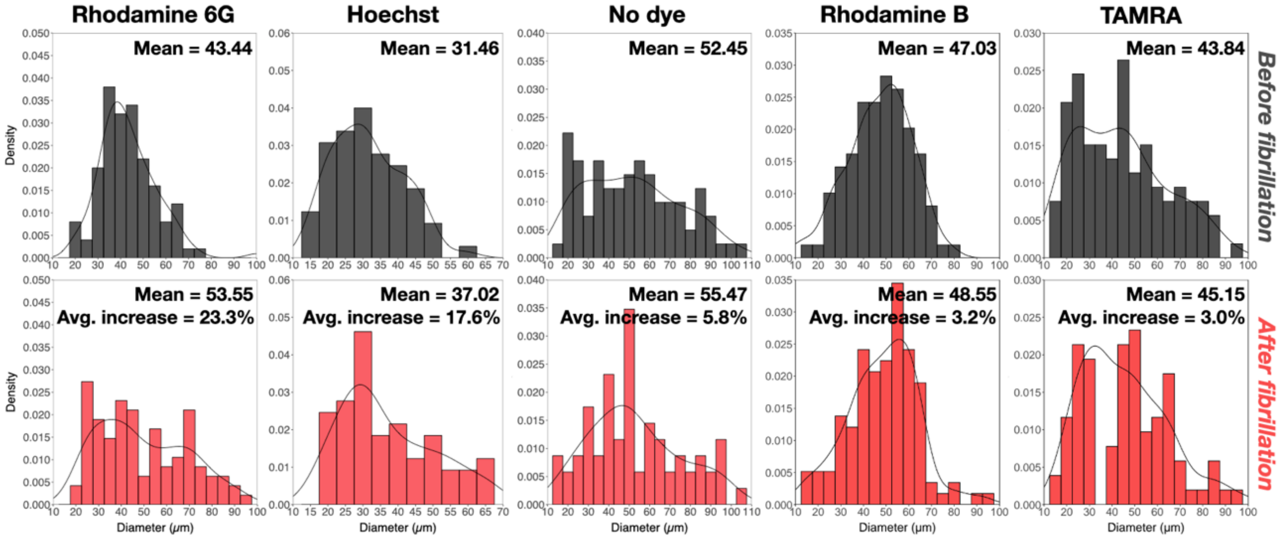

Supplementary Figure 10. Size distribution of droplets loaded with $\mathbf{P_{C8}}$ before (top row) and after (bottom row) addition of $\mathbf{T_8}$ –and subsequent fibrillation– in presence of different fluorescent dyes (see labels). The average increase ('Avg. increase') in droplet size -which is the value plotted in Fig. 3c (mean±SD)- represents the difference in mean size ('mean') after fibrillation divided by the mean size before fibrillation (%). *n*≥65.

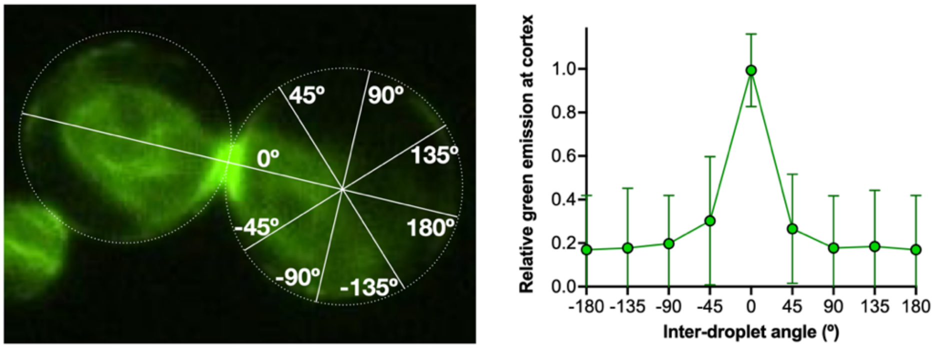

Supplementary Figure 11. Representative epifluorescence micrograph of two contacting droplets with their centres connected through a straight line (0°). Angles drawn from each droplet centre on either direction (see labels). The plot represents the average pixel intensity (green emission) at the cortex of the droplets measured at each of the angles indicated on the left; n=24(mean±SD). Note -180° = 180°.

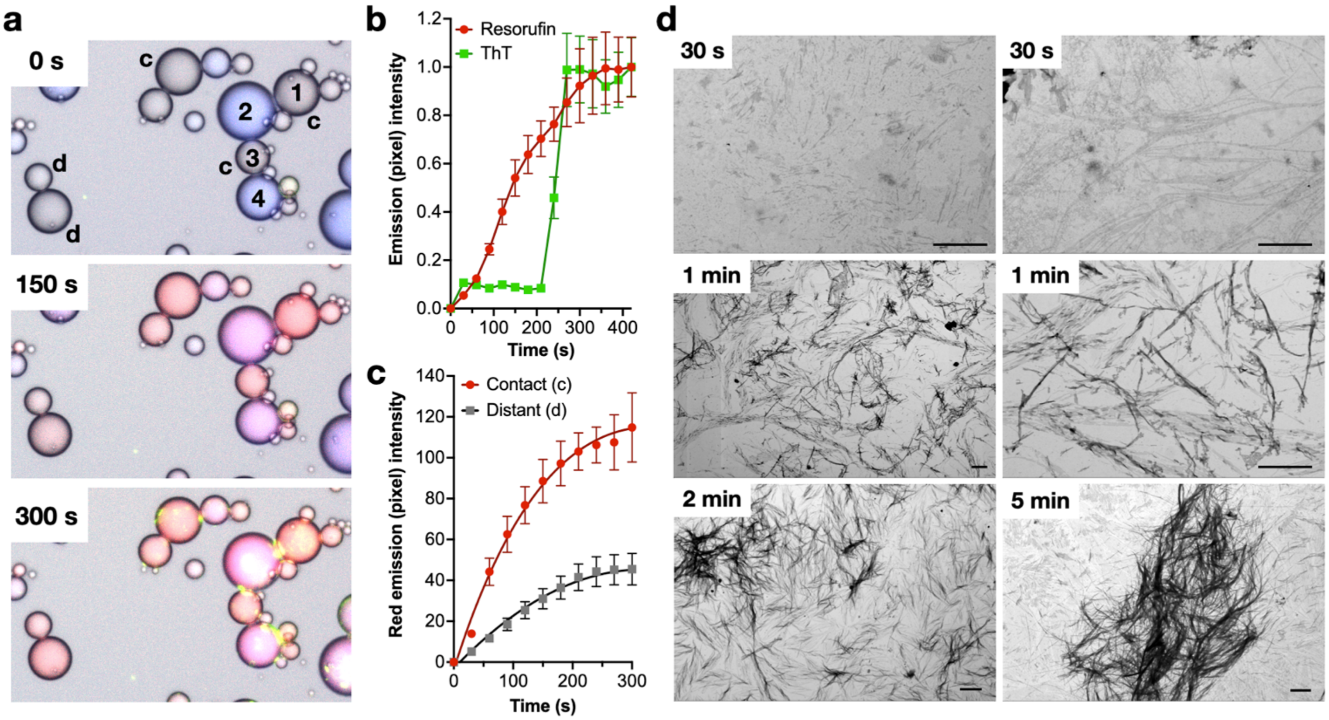

Supplementary Figure 12. (**a**) Epifluorescence time-lapse images of droplet populations containing either GOx (blue) or HRP (colourless), both loaded with $\mathbf{P_{C8}}$, after addition of octanal ($\mathbf{T_8}$) at t=0 s (see Movie 10b). Merged channels: GOx (blue), resorufin reporter (red), ThT-stained microfibres (green). (**b**) Average microfibre (ThT) and red (resorufin) emission intensity of droplets 1-4 (see labels) normalized to the maximum value observed for each channel; n=4(mean±SD). (**c**) Average red emission intensity of HRP droplets in contact (c) or distant (d) from GOx droplets – see labels; n=3 (mean±SD). (**d**) STEM images of $\mathbf{P_{C8}}$ in reaction with $\mathbf{T_8}$ evidencing rapid nanofibre production (30 s) with progressive bundling over time (1-5 min). Scale bars = 1 μm.

5. SUPPLEMENTARY REFERENCES

1. Miller, D. Dynamic Surface Tension: Industrial Applications and Characterisation of Commercial Surfactants*. *Tenside, Surfactants, Deterg.* **42**, 204–209 (2005).

2. Perlikowska, W. & Mikołajczyk, M. A Short Synthesis of Enantiomeric Phytoprostanes B1 Type I. *Synthesis* **16**, 2715–2718 (2009).
