# Supplementary material for: Supramolecular fibrillation of peptide amphiphiles induces environmental responses in aqueous droplets
Source: Nat Commun. 2021 Nov 5;12:6421. doi: 10.1038/s41467-021-26681-2 (PMC8571317; doi:10.1038/s41467-021-26681-2)
Supplement: Supplementary file 2 — Description of Additional Supplementary Files [file 41467_2021_26681_MOESM2_ESM.docx]

#

# supplementary movie LEGENDS

**Supplementary Movie 1**

Epifluorescence microscopy video showing the microfibre forming reaction in water, imaged using Thioflavin T (green) to help visualize the fibres. Total video length is 5 s and is shown at a real-time speed of x60 at 1 frame per second.

**Supplementary Movie 2**

Epifluorescence microscopy video showing the microfibre forming reaction in water-in-oil emulsion droplets, imaged using Thioflavin T (green) to help visualize the fibres. Total video length is 20 s and is shown at a real-time speed of x30 at 1 frame per second.

**Supplementary Movie 3**

Cropped video of Movie 2. Length of video, frames per second and real-time speed is the same as Movie 2.

**Supplementary Movie 4**

Confocal microscopy video of a z-stack taken of droplets containing microfibres after the reaction and self-assembly, imaged using Thioflavin T (green) to help visualize the fibres. The video consists of 43 slices taken in 10 µm increments and is shown at a speed of 5 frames per second.

**Supplementary Movie 5**

Cropped video of Movie 2. Length of video and recorded interval between each frame is the same as Movie 2 but the video itself is shown at 120x real-time speed.

**Supplementary Movie 6a**

Epifluorescence microscopy video showing the microfibre forming reaction in water-in-oil emulsion droplets and the subsequent uptake of Rhodamine 6G (red). Total video length is 13 s and is shown at a real-time speed of x60 at 2 frames per second.

**Supplementary Movie 6b**

Epifluorescence microscopy video showing the microfibre forming reaction in water-in-oil emulsion droplets and the subsequent uptake of Rhodamine 6G (red). The microfibres are imaged using Thioflavin T (green). Total video length is 10 s and is shown at a real-time speed of x60 at 2 frames per second.

**Supplementary Movie 7a**

Epifluorescence microscopy video showing the microfibre forming reaction in water-in-oil emulsion droplets and the subsequent uptake of Rhodamine B (red). Total video length is 15 s and is shown at a real-time speed of x60 at 2 frames per second.

**Supplementary Movie 7b**

Epifluorescence microscopy video showing the microfibre forming reaction in water-in-oil emulsion droplets and the subsequent uptake of Rhodamine B (red). The microfibres are imaged using Thioflavin T (green). Total video length is 9 s and is shown at a real-time speed of x150 at 5 frames per second.

**Supplementary Movie 8**

Epifluorescence microscopy video showing the microfibre forming reaction in water-in-oil emulsion droplets and the subsequent uptake of Hoechst (blue). The microfibres are imaged using Thioflavin T (green). Total video length is 10 s and is shown at a real-time speed of x60 at 2 frames per second.

**Supplementary Movie 9**

Epifluorescence microscopy video showing the microfibre forming reaction in water-in-oil emulsion droplets and the subsequent uptake of TAMRA (red). The microfibres are imaged using Thioflavin T (green). Total video length is 10 s and is shown at a real-time speed of x150 at 5 frames per second.

**Supplementary Movie 10a,b**

Epifluorescence microscopy video showing the microfibre forming reaction in water-in-oil emulsion droplets and the subsequent communication between two populations of droplets each containing one enzyme in a two-enzyme cascade. The microfibres are imaged using Thioflavin T (green). The separate enzyme populations are distinguished by GOx-DyLight 405 (blue) and the communication is demonstrated by the appearance of resorufin (red). Total video length is 10 s and is shown at a real-time speed of x30 at 2 frames per second. The two movies represent different areas of the same sample, with Movie 10a not showing the brightfield channel, whereas Movie 10b does include brightfield in the merged channels.

**Supplementary Movie 11**

Epifluorescence microscopy video showing the control experiment (without octanal=**T_8_**) of Movie 10. All other aspects of the video such as the length of video, frames per second and real-time speed is the same as Movie 10.
